# Identification of Human Walking Balance Controller Based on COM-ZMP Model of Humanoid Robot

**DOI:** 10.3389/frobt.2022.757630

**Published:** 2022-02-24

**Authors:** Taizo Yoshikawa

**Affiliations:** Honda R&D Co., Ltd., Wako, Japan

**Keywords:** walk assist device, balance control modeling of human, balance control of humanoid, COM-ZMP model, gait analysis, inverted pendulum, gait phase analysis, human movement analysis

## Abstract

The purpose of this research is to build a technology that enables wearable robotic systems that support human movement to maintain stable balance. By expanding our knowledge of conventional human gait analysis technology and robotics technology, we will build a technology that can estimate the state of human balance. In order to build a technology for estimating the human balance state based on the balance control technology of humanoid robots, we conducted joint research with Osaka University. We applied our knowledge of humanoid robot control to human stepping and braking motions, and confirmed the effectiveness of the balance control model using data measured by a motion capture system and a floor reaction force sensor system. In order to build a technology for estimating the human balance state based on the balance control technology of humanoid robots, we conducted joint research with Osaka University. We applied our knowledge of humanoid robot control to human stepping and braking motions to build a human balance control model. We confirmed the effectiveness of the balance control model using data measured by a motion capture system and a floor reaction force sensor system. In order to understand the state of human walking, the human walking motion was measured by motion capture and analyzed in detail. Following the norms of gait analysis techniques, we extended the balance control model of human foot-stepping and braking motions to a gait model that includes continuous straight-line walking and change of direction during walking. The effectiveness of the constructed balance control model was confirmed using a motion capture system and a floor reaction force sensor system.

## Background

Japan’s labor force is expected to decline year by year due to the aging population, declining population, and declining birthrate. According to a report by the Ministry of Health and Welfare, the number of employees was 62.7 million in 2012, but it is expected to be 60 million if economic growth and labor participation do not proceed properly in the future. In addition, simulations predict that the number of employees will decrease to 54.49 million in 2030. It is said that the declining birthrate and aging population will progress when one of the 2.5 people nationwide becomes an elderly person aged 65 or over in 2060. Considering this situation, it is important for companies to take a new approach to utilizing human resources in the future. In order to counter the declining workforce, it is necessary not only to increase the proportion of foreigners, but also to allow more women, the elderly and people with disabilities to participate in physically demanding jobs. In order to utilize diverse human resources, it is necessary to create an environment where employees can work comfortably and achieve results. In order to improve work efficiency and safety, it is necessary to actively complement exercise work in a production environment. It is necessary to evaluate the workload of standard ergonomic work, reduce high-load posture work, improve the process, and place it in the optimal process that matches the capabilities of the operator. In recent years, analysis based on kinematics models and musculoskeletal models based on robot technology is applied and has been incorporated. Physiological and psychological models are being incorporated into workloads as needed to perform ergonomic workload assessments. With the progress of computerization and the spread of new technologies such as AI and robotics, it has become possible to replace tasks that were difficult to mechanize with machines and systems. In order to build various production systems, the introduction of robot technology that physically supports the movement of workers in various specialized fields is attracting attention.

Research in robotics has drawn many ideas by capturing and imitating humans as a system. Human-mimicking approaches have been applied in the design of manipulators, sensors, actuators, etc., coordinated movements of the whole-body, complex tasks, and the realization of advanced action plans for interacting with the external environment and humans. Humanoid robots are thought to be able to support our daily life, social activities, and labor work in the same environment as humans, without significantly changing these environments. If it is a humanoid robot that has the same physique as a human and can move in the same way, tools and vehicles for human use can be used as they are. Humanoid robots are expected to be used in various situations such as assistance in our daily lives, assistance in the field of nursing care, production activities, and disaster support. In anticipation of such needs, various robots such as Waseda University’s “WABOT”, Honda’s “ASIMO”, Sony’s “QRIO”, Fujitsu’s “HOAP”, and “HRP” series of AIST developed by Kawata Robotics, have been made. Boston Dynamics of the United States is developing a quadruped walking robot “SPOT” and a humanoid robot “Atlas” with the support of DARPA (Defense Advanced Research Projects Agency). Toyota announces the full-body remote control robot T-HR3. The development of an avatar (alternate) robot is underway in which a humanoid robot performs the same movement by measuring and transmitting the movement and torque of a driver equipped with a skeletal control system.

**FIGURE 1:**
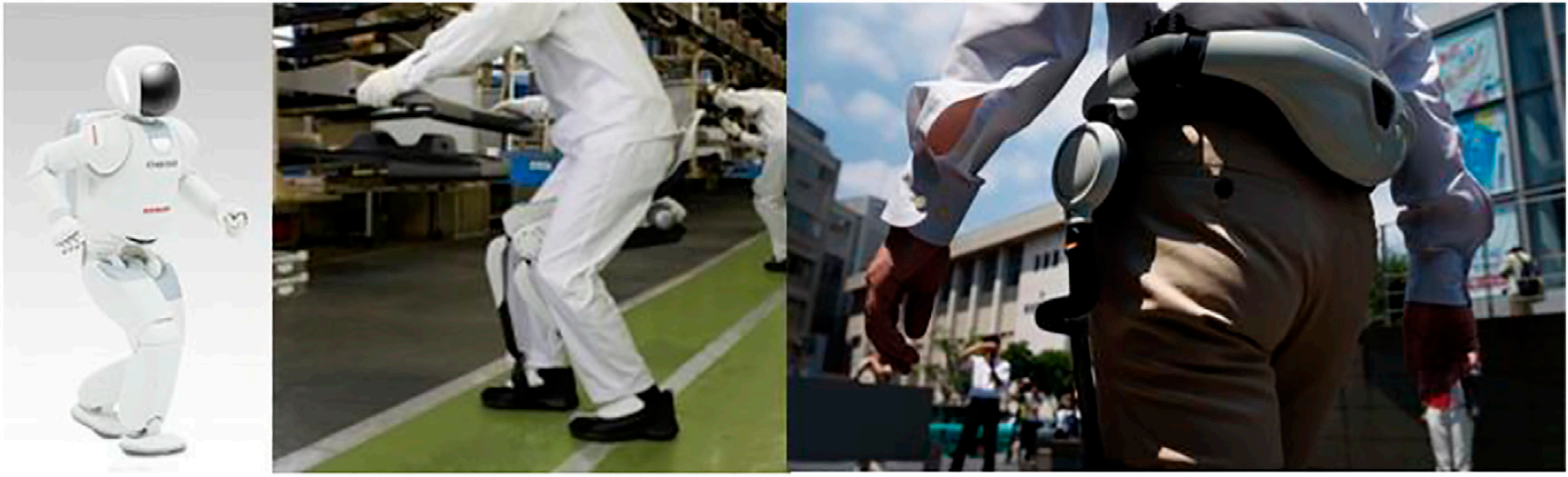
Robotics technology for motion assist and motion analysis.

Today, the methodologies developed and used in the field of robotics (Figure 1) are mature enough to address research agendas in many other areas, from neuroscience to computer animation. Techniques are attracting attention for using robotics techniques and control theory to gain basic insights into the natural movements of humans and to understand the mechanisms that lead to improved quality of treatment and rehabilitation. If technology in the robot domain is introduced in addition to the conventional human analysis technology, it will be possible to estimate and analyze the internal state during human movement. In addition, by introducing simulation technology, it will be possible to estimate and analyze the internal state of muscles and skeletons. For example, it has been difficult to estimate the internal state and balance state of the musculoskeletal level when a person is walking, but it is considered that it will be possible by introducing the technology constructed in the robot domain.

**FIGURE 2:**
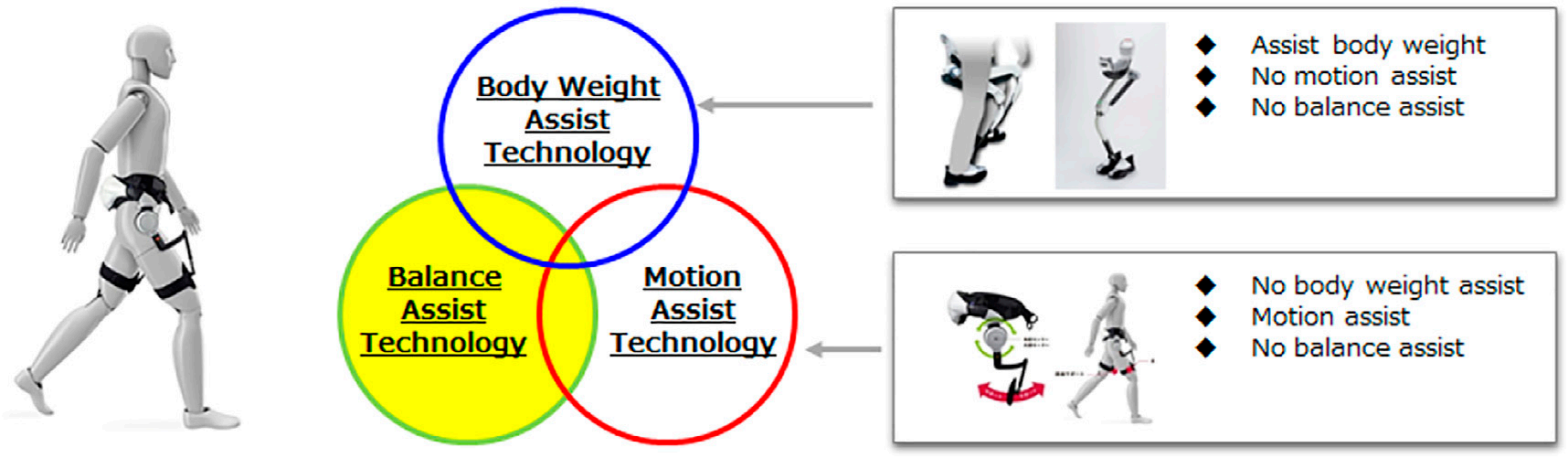
Category of motion assist and motion technology.

Conventionally, robot technology that assists human movements has focused on the function of supporting weight and reducing the load, and the function of recovering movements by assisting movements such as walking, and various devices have been developed (Figure 2). On the other hand, when the wearer wears the assist device and operates, the balance is not assisted in most cases. Therefore, the balance may be lost during operation, which is not a safe condition for the wearer. Further, even if there is a function of simply assisting the balance, if the balance state of the wearer cannot be estimated accurately, a mismatch with the balance of the wearer will occur, and the system will not be safe for the wearer. In order to compensate the wearer’s balance in the assist device, it is necessary to constantly estimate the wearer’s balance state. At the same time, it is important to predict the short-term future condition and recover to the optimum balance condition before the balance condition becomes unstable. In this study, we construct a balance control model for predicting short-term future states, and a balance observer that enables us to estimate and predict balance states based on the deviation between the predicted balance state and the continuously estimated balance state. In this paper, we discuss the construction of a balance control model.

## Human Gait Understanding

**FIGURE 3:**
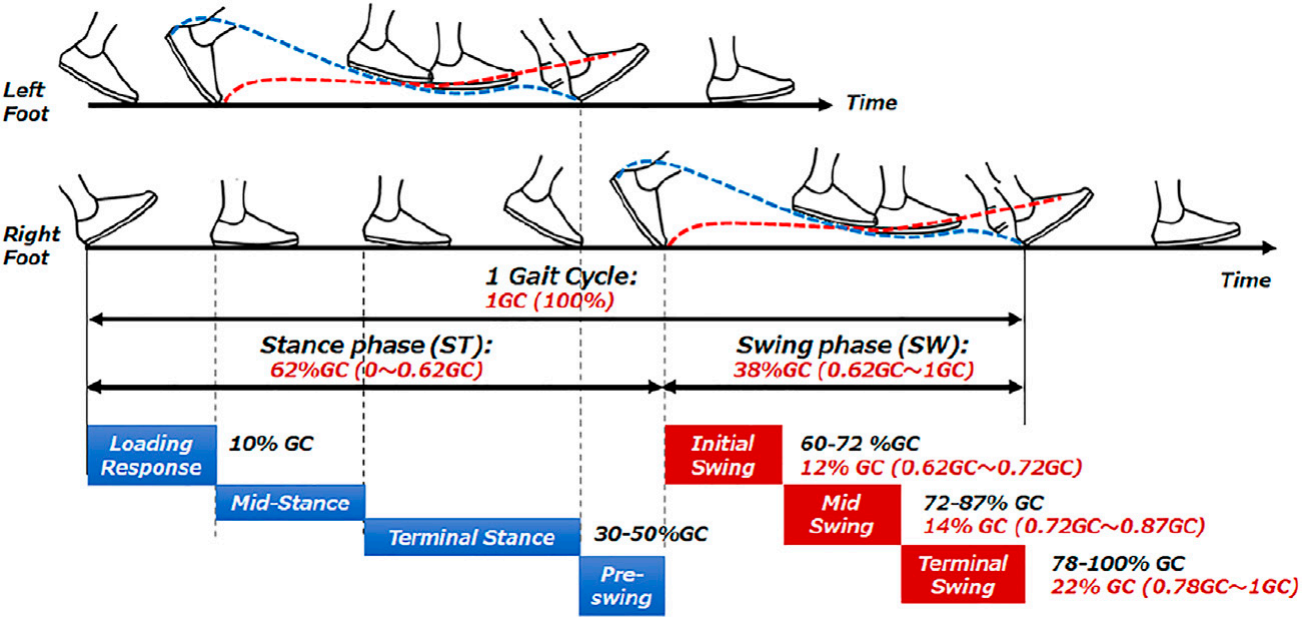
Human gait timing.

Traditionally, improvement of gait movement has been done by analyzing gait through observation based on the experience of physical therapists or by using equipment. Movement analysis by physical therapists has been done based on experience and intuition, focusing on the patient’s joint movements and body balance. Based on the experience of the physical therapists, it has been thought to organize what kind of activities can be seen in each phase of the stance phase and walking phases. In this section, we first summarize the events that determine the gait cycle and their names. The events that determine the gait cycle and their names are listed below and is shown in Figure 3. In order to understand gait, Perry’s gait analysis (Perry, 2010) was used as a reference. In Perry’s gait analysis, the walking body is divided into two parts: the boarding part consisting of the head, upper limbs, trunk, and pelvis, and the motor part consisting of the pelvis and lower limbs. Walking was defined as a repetitive motion with two steps as one gait cycle, and each state during one gait cycle was defined. Focusing on one leg, the period when the foot is on the floor is called the stance phase, and the period when the foot is not on the floor is called the swing phase. When focusing on both feet, the period in which both feet are on the floor is defined as the bipedal support phase, and the period in which only one foot is on the floor is defined as the unipedal support phase. In the case of normal walking, the timing at which the lower leg of the subject’s foot becomes vertical coincides with the timing at which the heel of the opposite foot floats. In the case of normal walking, the load response period of the subject’s foot corresponds to the anterior swing period of the opposite foot.

Assuming that one cycle from heel contact to the next heel contact is 100%, the average walking cycle is 62% in the stance phase and 38% in the swing phase. As the walking speed increases, the swinging time generally decreases and the speed increases.

### Initial Contact

The moment the subject’s foot touches the floor.

### Load Response

The period from the timing of the first contact to the time when the opposite foot leaves the floor is called the load response period. At this time, the entire sole of the foot touches the floor with the heel as the center of rotation. A reaction force from the ground is generated on the back side of the foot, centered on the heel, and the floor reaction force moves from the back side to the front side of the foot palm. During the load response period, the heel contact causes the flexible tissue of the heel to absorb the contact impact, and the effective contraction of the dorsiflexor muscles absorbs the contact impact. The knee is pulled forward and a small flexion occurs in the knee joint, causing the reaction force from the ground to pass behind the knee and generate the force to flex the knee. After the first contact with the ground, the ground reaction force increases sharply, reaching almost 100% of the body weight when the entire sole of the foot touches the surface. At the end of the load response period, the ground reaction force will be 120% of body weight. The ground reaction force at this time is a vector from the center of load of the foot toward the center of gravity. Therefore, from the initial stage of ground contact to the end of the load response period, the ground contact reaction force becomes backward and the whole-body acts as a brake. On the other hand, although the target foot is braked by touchdown, the center of gravity moves forward with the whole-body centering on the support leg, and the position of the center of gravity is accelerated in the direction of movement by generating a rotational moment. That is the trigger that generates the next step.

### Mid-Stance

In Mid-Stance, the knees return from a slightly bent state to a stretched state, and the entire sole of the foot touches the ground and stabilizes. In the first half of Mid-Stance, the upper body and lower limbs rotate forward around the ankle joint, and the center of gravity is pushed up. On the opposite leg, the lower limbs are swung forward. In normal walking, the activity of the extensor muscles and the action of the refracting muscles cancel each other out, so that the trunk can be kept stable and vertical. The ground reaction force generated on the heel after landing moves to the forefoot side by moving the upper body forward. When the center of gravity is highest, the ground reaction force goes straight up. In the latter half of Mid-Stance, the center of gravity moves forward with the ankle joint as the center of rotation, so the ground reaction force tilts forward and accelerates forward. The continuous movement of the center of gravity makes the ankle joint move smoothly, and the movement of the ground reaction force becomes smooth. Therefore, the ground reaction force is directly linked to the movement of the center of gravity. While walking, the position of the center of gravity bends to the left and right by exchanging the left and right support legs, and acceleration and deceleration in the front-back direction is repeated by braking operation by heel landing and forward acceleration by swing operation. The upper body is kept vertical without bending sideways or tilting back and forth. The state of walking is determined by the relationship between the position of the center of gravity and the support basal plane formed by the support legs. After Mid-Stance, the ground projection point of the center of gravity exists inside the support basal plane in the two-leg support period, but the ground projection point of the center of gravity is outside the support basal plane in the one-leg support period, and it is in an unstable state.

### Terminal Stance

When the position of the center of gravity of the whole body moves forward and the heel on the back side floats, it shifts from the middle stage of stance to the end stage of stance. The characteristics of the movement during this period are to bend the body backward, adjust the landing position and timing of the opposite foot, and limit the descent speed of the opposite foot so as to suppress the landing impact of the initial contact.

### Pre-Swing

The state in which the opposite foot first touches the ground and the whole body is supported by both feet. The whole body is supported by both feet, and the ground reaction force of the foot that was the supporting leg decreases, and at the same time, the ground reaction force of the opposite foot increases. The ground reaction force of the subject’s foot is tilted forward before the opposite foot first touches the ground. On the other hand, the foot on the opposite side is braked with the ground contact reaction force tilted backward at the same time as the initial touchdown. As the ground contact reaction force of the opposite foot increases, the ground contact reaction force that was tilted forward gradually decreases.

### Initial Swing

At the same time as the subject’s foot takes off from the ground, the knee bends due to the inertial force of the lower limbs. The initial stage of the initial swing is until the target foot overtakes the opposite foot.

### Mid-Swing

The subject’s foot overtakes the opposite foot, bending the hip joint and pulling the knee forward. When the subject’s feet are at knee height so that they do not touch the floor, extend the knees. The period until the lower leg is perpendicular to the ground is called the Mid-Swing period.

### Terminal Swing

In normal walking, the flexors of the knee contract efficiently, and the knee suddenly stretches and brakes. The timing when the knee finishes stretching coincides with the timing when it first touches the ground.

## Experimental and Measurement Environment

### Experimental Environment

**FIGURE 4:**
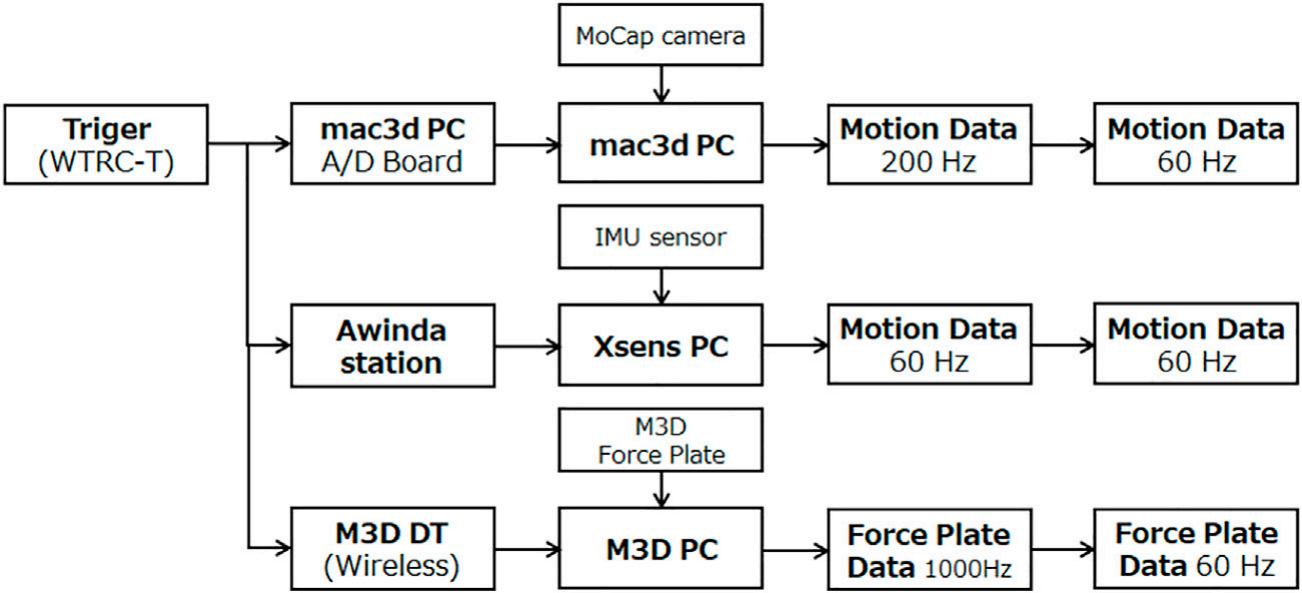
Measurement system.

**FIGURE 5:**
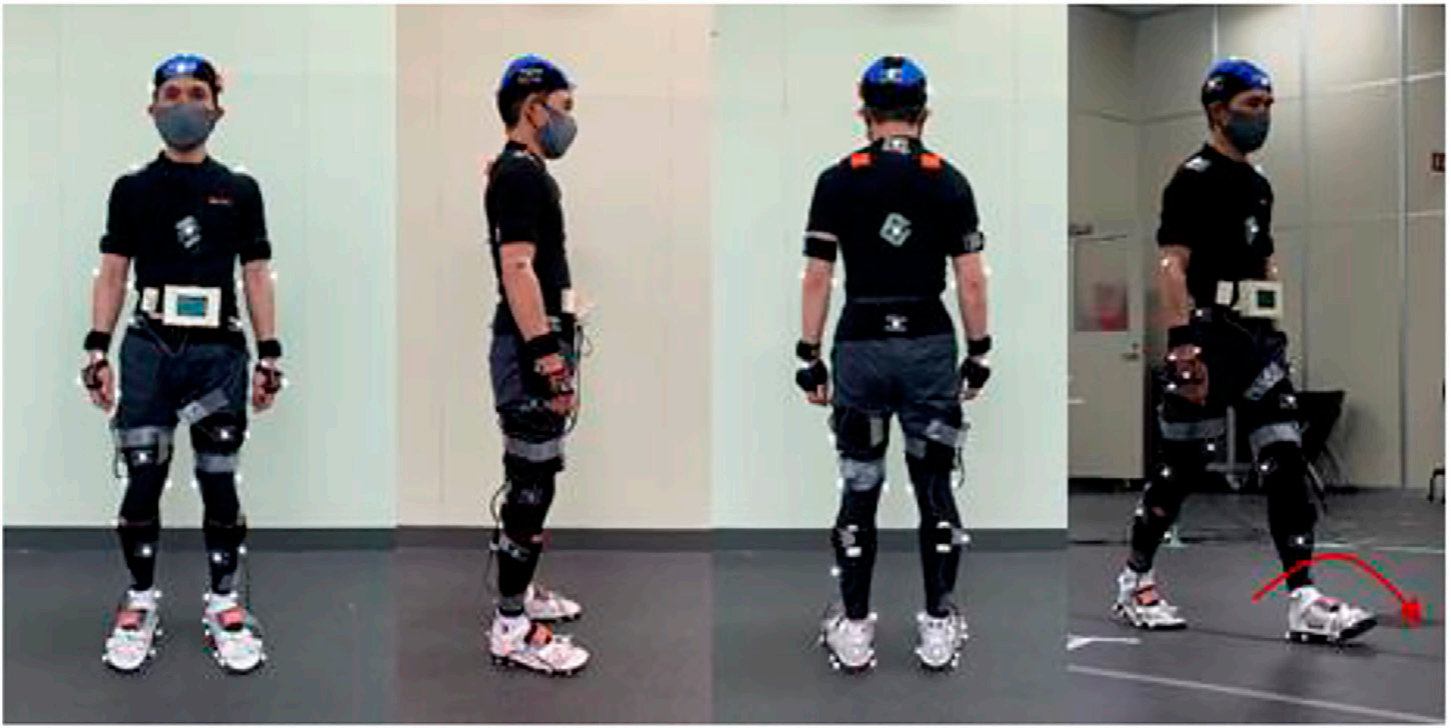
Installation status of measurement system.

Based on early experiments and discussions, we assumed that we would measure continuous walking data, and used a wearable measurement system that can measure over a wide range. For gait measurement, Xsens motion capture system^2^ and a force plate M3D (manufactured by Tech Gihan Co., Ltd.)^1^ were used. Xsens is a highly accurate and secure PC-based sensing application development platform consisting of 10-axis IMU sensors, receiver, measurement software (MT MANAGER), and software development kit (SDK). It is a system that can measure three-dimensional movements in the living environment and work environment and is used in various situations such as sports and rehabilitation. M3D is an ultra-thin 6-component force plate designed to be attached to shoes. There is M3D-EL-FP-W95 (W90 x D80) for toes and M3D-EL- for heels. The FP-W80 (W80 x D80) is combined to form four units with both feet. Inertial sensors (acceleration, gyro, geomagnetic) are integrated in each Force Plate to measure simultaneously with six force components. The wireless communication unit makes it possible to measure continuous ground reaction force in free space. The trigger signal was sent from the circuit board using the Xsens and the force plate M3D analog input terminal, and the Xsens and the force plate M3D were set to synchronize at the timing of recording start. The measured data was converted to 60 Hz after the measurement according to the frequency of Xsens. An optical motion capture system was also measured at the same time to verify the measurement data of Xsens, but the data was not used in this gait analysis and balance control model identification. The constructed measurement system is shown in Figure 4 and the situation of a subject wearing a measurement sensor is shown in Figure 5.

### Walking Data Measurement

**FIGURE 6:**
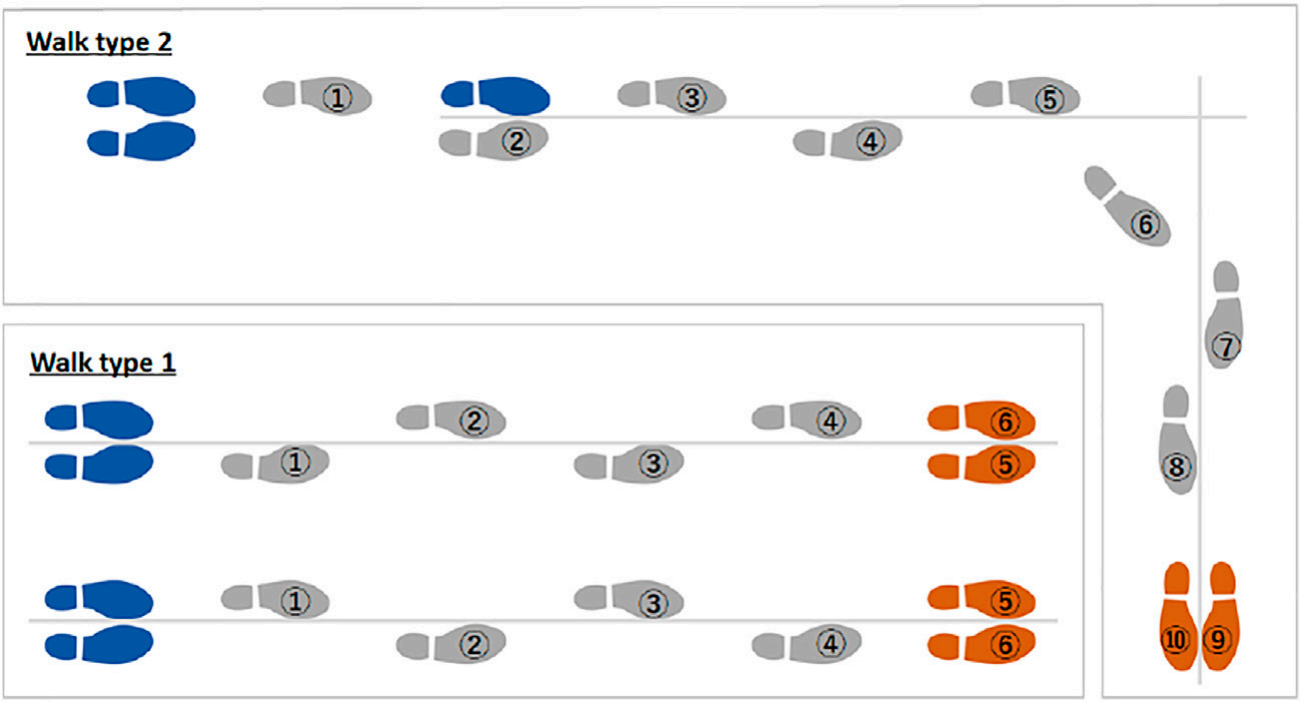
Measured walking pattern.

Figure 6 shows the measured walking pattern. A motion capture system and a force plate were attached to the subject, and two walking data were measured: 1) going straight, and 2) going straight, turning, and going straight. In the straight-ahead line, six steps were taken from the start of walking, but the walking speed and stride of walking were not limited, and the subject was allowed to walk naturally.

For straight-ahead/change-direction/straight-ahead walking, the start position, change direction position, and stop position are set so that the pedestrian can walk naturally without limiting the walking speed and stride of walking. The walking movements of (1) and (2) were measured 10 times for each subject. The data of each measurement system obtained by the measurement was performed offline, the timing of the start trigger was synchronized, and the frequency was converted. Using the values of foot position (X, Y, Z), foot orientation (Quaternion), ankle position (X, Y, Z) and ankle orientation (Quaternion) measured by Xsens, the three-dimensional foot shape model is converted to and updated sequentially. For the force plate value of the M3D sensor, the value converted by the IMU sensor mounted on the M3D sensor as the value in the global coordinate system was used.

### Subject Body Shape Feature Modeling

The subject information is shown below. We thoroughly examined the ethical points regarding the experiment and the handling of personal information regarding the measurement data in advance. Three subjects were measured: 1) height 1.65 [m], weight 57 [kg], lean type, 2) height 1.60 [m], weight 55 [kg], lean type, 3) height 1.82 [m], weight 62 [kg], lean type. Since the physiques of subject 1) and subject 2) are almost the same, it is assumed that similar analysis results can be obtained. In addition, it was assumed that subject 3) was about 0.2 [m] taller and could be different from other subjects due to the difference in height. The height of subject 1, subject 2, and subject 3, the length of each segment, the vertical direction of the center of gravity, the weight, the weight of each segment, and the distribution ratio of the center of gravity are shown in Table 1. Geometric connection structure, joint rotation direction and length, center of gravity position, and weight of each segment were defined in URDF format, which is commonly used in robots.

**TABLE 1:** Segment length and segment weight of subjects.

		Subject1		Subject2		Subject3		GC ratio γ
		Height [m]	Weight [kg]	Height [m]	Weight [kg]	Height [m]	Weight [kg]	
Whole-Body		1.65	57.00	1.60	52.00	1.82	62.0	ー
Segment	1) Head	0.22	3.93	0.21	3.59	0.24	4.3	0.821
	2) Chest	0.45	17.21	0.44	15.70	0.49	18.7	0.428
	3) Body	0.45	10.66	0.44	9.72	0.49	11.6	0.609
	4) Right Upper Arm	0.25	1.54	0.25	1.40	0.29	1.7	0.529
	5) Right Fore Arm	0.25	0.91	0.25	0.83	0.29	1.0	0.415
	6) Right Hand	0.17	0.34	0.16	0.31	0.18	0.4	0.891
	7) Left Upper Arm	0.25	1.54	0.25	1.40	0.29	1.7	0.529
	8) Left Fore Arm	0.25	0.91	0.25	0.83	0.29	1.0	0.415
	9) Left Hand	0.17	0.34	0.16	0.31	0.18	0.4	0.891
	10) Right Upper Leg	0.33	6.27	0.31	5.72	0.36	6.8	0.475
	11) Right Lower Leg	0.33	2.91	0.31	2.65	0.36	3.2	0.406
	12) Right Foot	0.10	0.63	0.10	0.57	0.11	0.7	0.595
	13) Left Upper Leg	0.33	6.27	0.31	5.72	0.36	6.8	0.475
	14) Left Lower Leg	0.33	2.91	0.31	2.65	0.36	3.2	0.406
	15) Left Foot	0.10	0.63	0.10	0.57	0.11	0.7	0.595

**TABLE 2:** Algorithm for gait phase and timing.

Phase	Start timing	End timing
Initial contact	Timing when the position of the lowest point of the foot shape model approaches the floor surface	
	Timing when the vertical value of the M3D heel side sensor changes from the value near 0 to the plus side	
Loading Response	End timing of Initial Contact	The position of the highest point of the foot shape model is smaller than a certain threshold value from the floor surface
		Timing when the vertical value of the M3D toe sensor changes from the value near 0 to the plus side
Midstance	End timing of Loading Response	The position of the highest point of the foot shape model is larger than a certain threshold value from the floor surface
		Timing when the vertical value of the M3D heel side sensor changes from a positive value to near 0
Terminal Stance	End timing of Midstance	Timing when the position of the lowest point of the foot shape model on the opposite side approaches the floor surface
		Timing when the value in the vertical direction of the sensor on the opposite side of the M3D heel changes from the value near 0 in the swing phase to the plus side
Pre-Swing	End timing of Terminal Stance	The position of the lowest point of the foot shape model is larger than a certain threshold value from the floor surface
		Timing when the vertical value of the M3D toe sensor changes from a positive value to near 0
Initial Swing	End timing of Pre-Swing	Timing when the position of the ankle of the target foot coincides with the position of the ankle of the opposite foot with respect to the direction of travel
Mid-Swing	End timing of Initial Swing	Timing when the knees of the target foot and the ankles are aligned on the horizontal plane or within the threshold range
Terminal Swing	End timing of Mid-Swing	Timing when the position of the lowest point of the target foot shape model approaches the floor surface
		Timing when the vertical value of the target M3D heel side sensor changes from the value near 0 in the swing phase to the plus side

**FIGURE 7:**
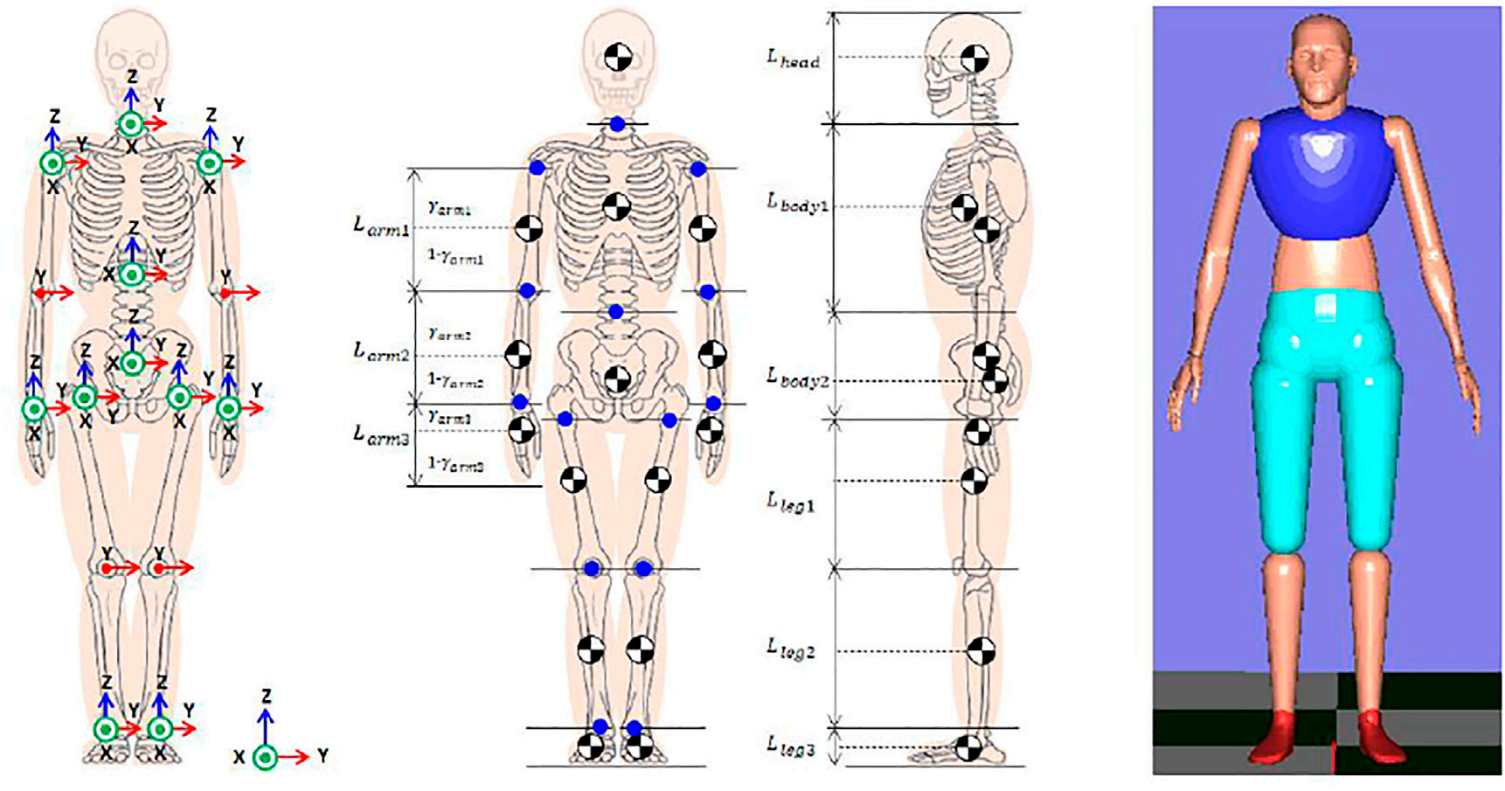
Subject body shape feature modeling.

The human body is supported by a combination of 200–300 bones. However, it is difficult and inefficient to model the skeleton of the whole body in detail. We defined a model of joints of the whole body with the joint structure as shown in Figure 7 based on the knowledge of the whole-body control of humanoid robots. The whole body is regarded as a link system consisting of 15 segments of head, upper torso, lower torso, left and right upper arms, forearms, hands, thighs, lower legs, and feet as shown in Figure 7. In addition, since this model is equivalent to the Japanese body statistical model (Saito, 2015)^2^, we used the rigid body characteristic constants of (Ae et al., 1992). In the Japanese body statistical model, the internal division ratio that determines the position of the center of mass is given from the average mass distribution model. After identifying the mass for each segment, the center of mass position for i = 1 to 15 segments in local coordinate system were calculated by the following formula.

$$P_{G\left[i\right]}=\left(P_{Joint\left[i+1\right]}-P_{Joint\left[i\right]}\right)\gamma$$ (1)

**FIGURE 8:**
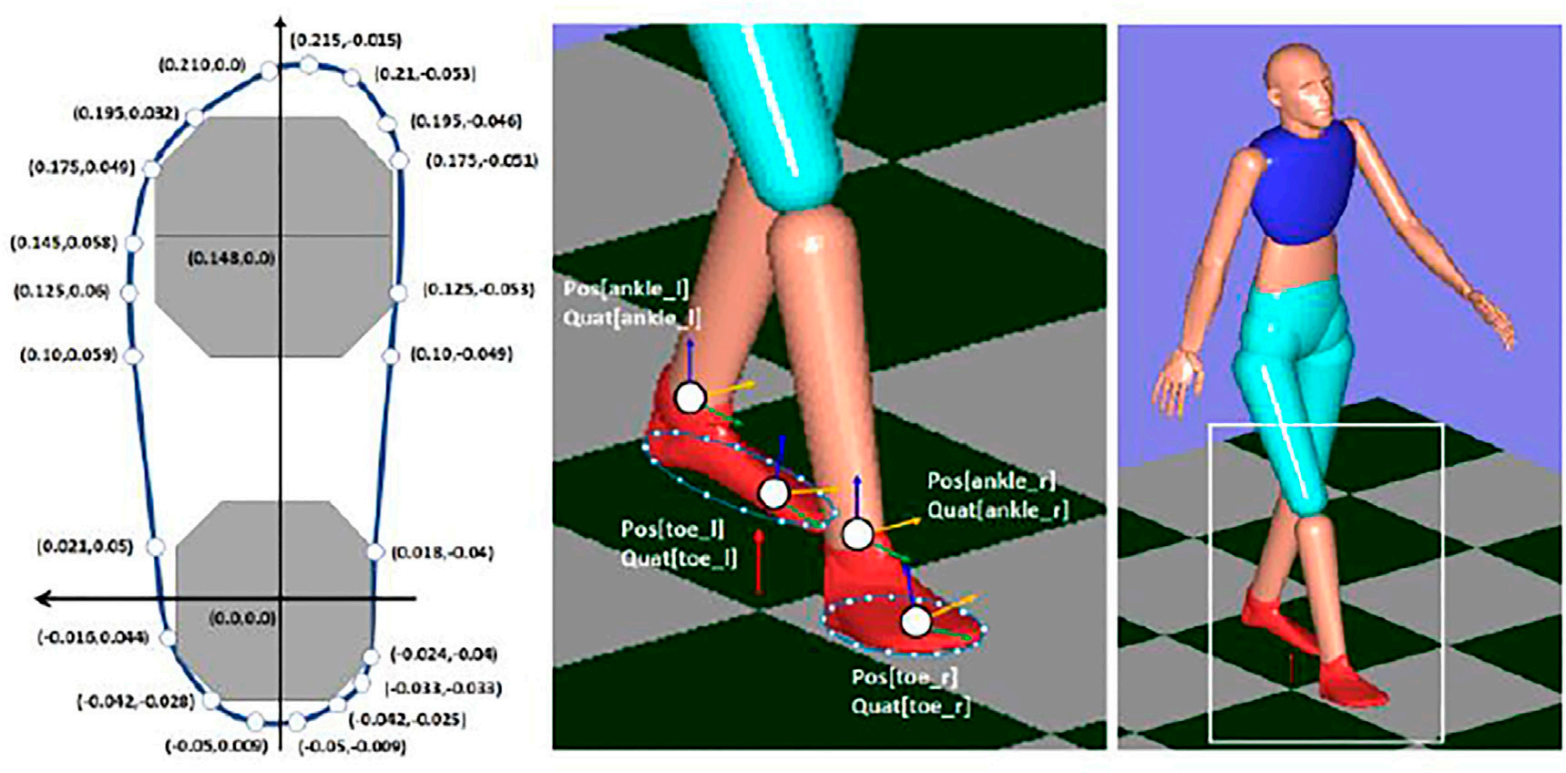
Subject foot shape feature modeling.

The M3D sensor is mounted on the provided insole, and the subject is wearing the M3D-mounted insole. By modeling the shape of the insole and using the 3D position and orientation information from Xsens, it is possible to update the position of each point on the insole online. By calculating the distance from the floor surface at each point of the insole, it is possible to estimate the ground contact condition between the insole and the floor. The shape of the insole was modeled as shown in Figure 8.

### Development of Gait Phase Detection Algorithm

**FIGURE 9:**
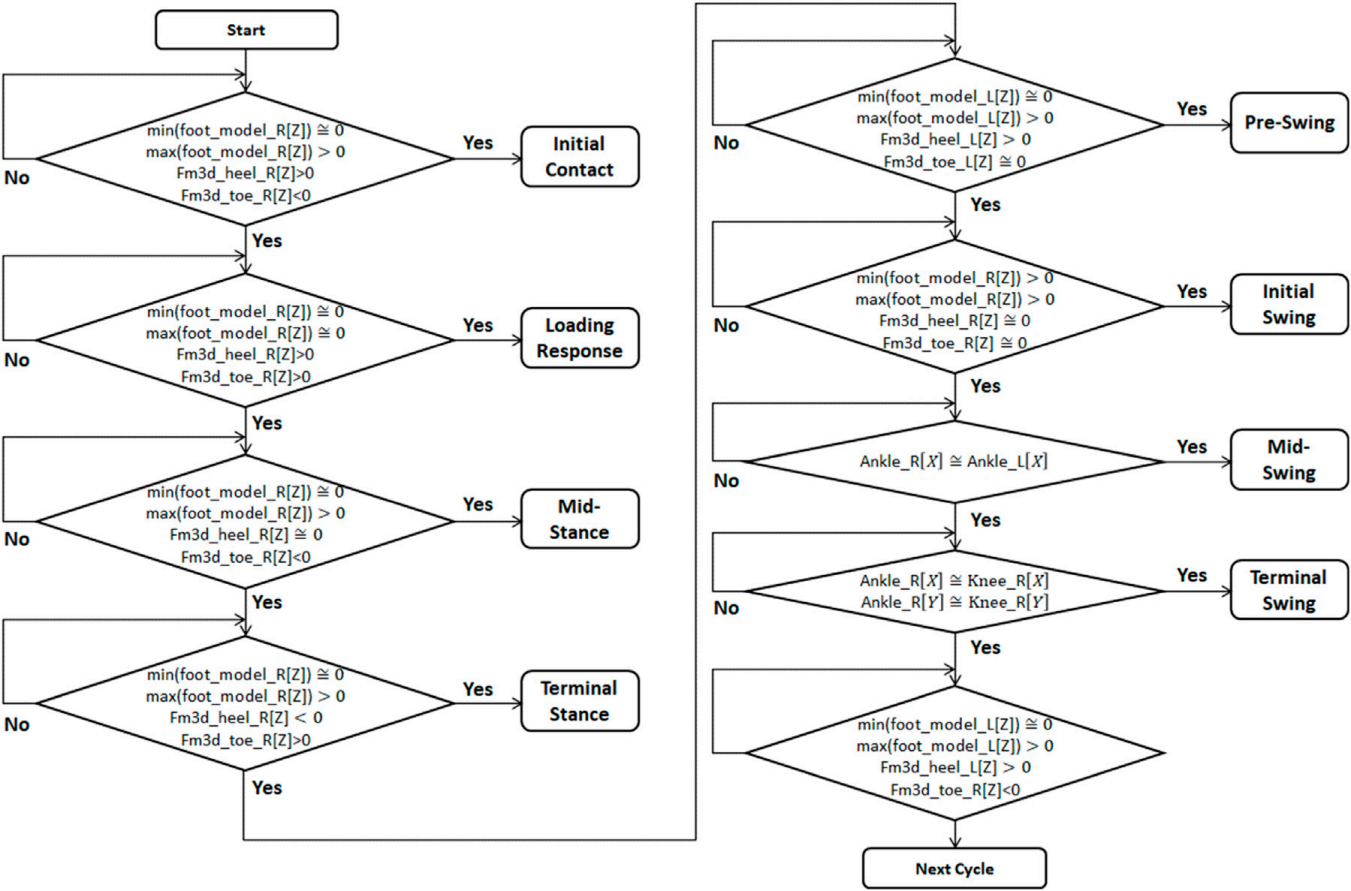
Algorithm to identify Gait phase while walking.

We constructed a system to identify each gait phase. By setting the following timing, a system for distinguishing each phase was constructed. Figure 9 shows the algorithm for distinguishing each phase using the data of the Xsens motion capture system and the M3D force plate system.

### Analysts of Straight Forward Walk Motion Data

**FIGURE 10:**
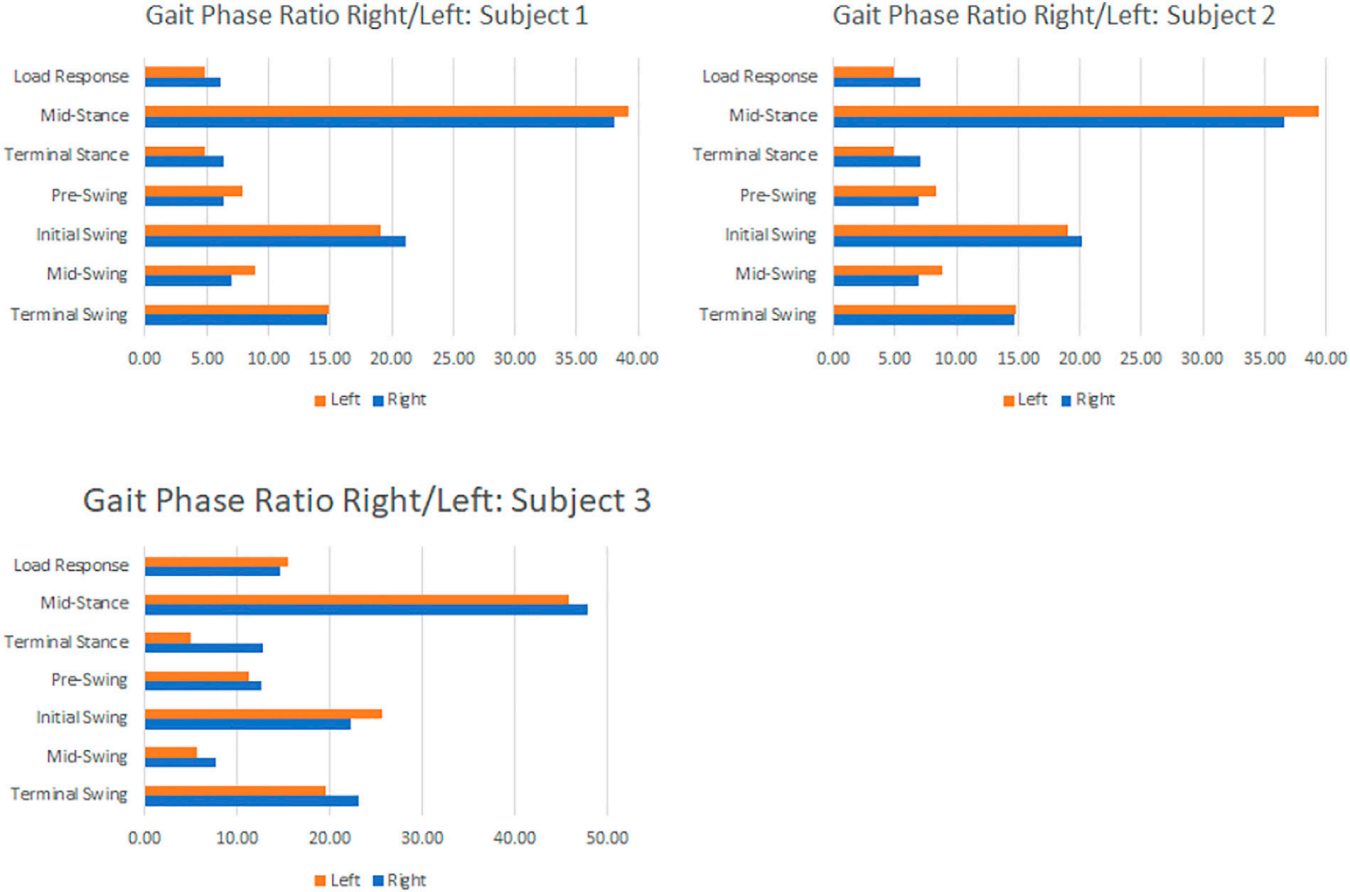
Human Gait ratio of each subject.

The data of straight-line walking is shown in Figure 10. Figure 10 shows the results of identifying each gait phase for left and right from the measurement data of subject 1, subject 2, and subject 3. The timing of each phase is shown when one Gait Circle is set to 100%. The seven defined gait phases were detectable by programming in the continuous walking state except for the timing of the start and braking of walking. As a result of the analysis, it was confirmed that all the subjects had different proportions of the right and left gait phases and the left and right strides. The proportion of each gait phase and the distribution of the stance phase and the swing phase also differed slightly between the left and right sides. This may be due to the fact that, unlike humanoid robots, the posture of humans is slightly different from that of left and right.

**FIGURE 11:**
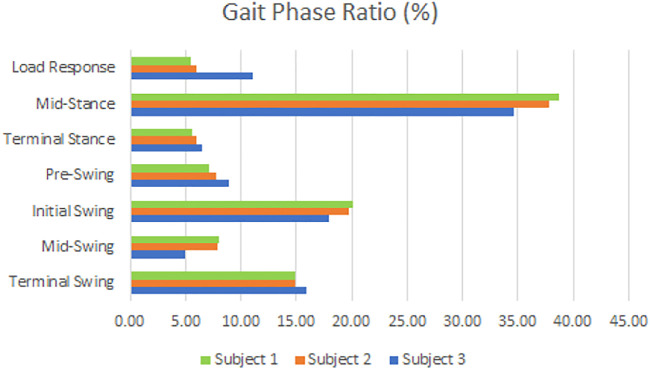
Comparison of gait phases of subjects.

Figure 11 compares the average values of each gait phase for subjects 1, 2, and 3. Subject 1 and subject 2, who are close to each other in height, have similar proportions of gait phase. In addition, the percentage of gait phase was different between subjects 1 and 2 with the same height and subject 3 with a higher height. In general, a taller person is considered to have a longer stride width when walking. However, this could not be.

confirmed from the results of this gait measurement. The difference in stride length is thought to be dominated by factors related to walking style rather than height. Although the difference in height seems to have a slight effect on the ratio of each gait phase, it cannot be determined with the number of three subjects. Even if the difference in height affects the ratio of each gait phase and changes the distribution between the stance phase and the swing phase, it is considered possible to switch between the stance phase and the swing phase by detecting heel contact and to consider the balance state. Therefore, we constructed an algorithm for estimating the human balance state based on the assumption that it is reasonable to focus on the horizontal component when considering the balance state.

## Balance Control Technology Based on Inverted Pendulum Model

The purpose of this research is to realize a stable balance maintenance function in the motion assist system. We are aiming for a technology that can estimate the balance state and guide it to the optimum balance state before the balance state becomes unstable. The purpose is to mathematically model human standing stability motion by utilizing the knowledge of balance control technology of humanoid robots based on the relationship between the center of gravity and the center of ground reaction force. The inverted pendulum model commonly used in balance control of humanoid robots uses the planned zero moment point (ZMP: zero moment point) (Harada et al., 2006; Sugihara and Nakamura, 2006; Takenaka, 2009; Takenaka et al., 2011) trajectory to generate a stable gait pattern. The following effects can be considered for the whole-body motion control of humanoid robots using ZMP.

A) It is possible to directly consider the physical constraints determined by contact with the ground.
B) The dynamic movement of a humanoid robot can be realized by the same principle as a general inverted pendulum model.
C) It is possible to consider only the global dynamics without considering the local dynamics of each link.

### Balance Phase Based on Inverted Pendulum Model

The exact dynamics of the humanoid robot is expressed by the following equation of motion (Sugihara and Nakamura, 2006).

$$\left[\begin{array}{cc} A_{11} & A_{12}\\ A_{21} & A_{22} \end{array}\right]\left[\begin{array}{c} {\ddot{q}}_{0}\\ \ddot{\theta } \end{array}\right]+\left[\begin{array}{c} b_{1}\\ b_{2} \end{array}\right]+\left[\begin{array}{c} 0\\ g \end{array}\right]=\left[\begin{array}{c} 0\\ \tau \end{array}\right]+\overset{N}{\underset{k=1}{\sum }}\left[\begin{array}{cc} K_{k11} & K_{k12}\\ K_{k21} & K_{k22} \end{array}\right]\left[\begin{array}{c} f_{k}\\ n_{k} \end{array}\right]$$ (2)

Here, ${\ddot{q}}_{0}$ is a six-dimensional acceleration including translation and rotation of the base link (trunk link) that is not fixed to the inertial system, θ is the joint angle vector, $A_{ij}$ is the inertial matrix, and $b_{i}$ is the centrifugal.

force and Coriolis force, $g_{i}$ is the gravity term, τ is the joint drive torque, N is the number of contact points between the robot and the environment, $f_{k}$ and $n_{k}$ are the external forces and moments acting at the k-th contact point, and $K_{kij}$ is the matrix that converts the external force into a generalized force. In this definition, there is no generalized force that directly drives the 6 degrees of freedom of the base link. Through the interaction with the environment caused by whole body movement, contact with the environment occurs, and the 6 degrees of freedom of the basal link are determined by the external force f_k and the moment n_k generated at the contact point between the robot and the environment. It can be seen that it is not effective to accurately derive the joint torque τ in real time by calculating the dynamics of the whole body in consideration of the external force $f_{k}$ and the moment $n_{k}$ generated by the contact of each link with the local environment. By focusing on the dynamics of the center of gravity, extracting the part related to translational motion from the upper part of Eq. (2) where the driving force is 0, and focusing on the global motion of the entire system, it is possible to effectively obtain a simple equation. it can. Expressed by the center of gravity of the entire robot ${\ddot{p}}_{G}$ , the sum of the external forces acting on the entire system $f={\left[\begin{array}{ccc} f_{kx} & f_{ky} & f_{kz} \end{array}\right]}^{T}$ , and the gravitational acceleration vector $g={\left[\begin{array}{ccc} 0 & 0 & g \end{array}\right]}^{T}$ ,

$$m\left({\ddot{p}}_{G}+g\right)=f$$ (3)

$$\overset{n-1}{\underset{i=0}{\sum }}m_{i}\left({\ddot{p}}_{G,i}+g\right)=\overset{N}{\underset{k=1}{\sum }}f_{k}$$ (4)

In Eq. 4, n is the number of links with the base link as link 0, $m_{i}$ is the mass of link i, $p_{G,i}={\left[\begin{array}{ccc} p_{Gx,i} & p_{Gy,i} & p_{Gz,i} \end{array}\right]}^{T}$ is the center of gravity position vector of the link i, and $f_{k}$ is the external force vector acting at the kth contact point. Focusing on the global motion of the entire system and extracting the part related to the rotational motion from the upper part of Eq. (2), the moment due to the external force received from the environment is as follows.

$$\dot{L}\left(p\right)=\left(p_{ZMP}+p_{G}\right)\times f+n_{Z}$$ (5)

Here, $\dot{L}\left(p\right)$ is the angular momentum acting around the center of gravity, $p_{ZMP}$ is the position vector of ground reaction force center (ZMP: Zero Moment Point) on the ground (ZMP) position vector $p_{ZMP}={\left[\begin{array}{ccc} x_{ZMP} & y_{ZMP} & z_{ZMP} \end{array}\right]}^{T}$ , $p_{G}$ is the position vector of the center of gravity of the whole body in the inertial coordinate system $p_{G}={\left[\begin{array}{ccc} x_{G} & y_{G} & z_{G} \end{array}\right]}^{T}$ , $n_{Z}$ are the total external force moments received from the environment around $p_{ZMP}$ , $n_{Z}={\left[\begin{array}{ccc} 0 & 0 & n_{Z} \end{array}\right]}^{T}$ . L(p) is the angular momentum that works around the center of gravity. Assuming that this change in angular momentum is sufficiently small and negligible, and that the change in the height of the center of gravity is negligible, the following approximate equation can be derived from Eqs 4, 5.

$$\ddot{x}=\zeta^{2}\left(x-x_{ZMP}\right)$$ (6)

$$\zeta =\sqrt{\frac{\ddot{z}+g}{z-z_{ZMP}}}≅\sqrt{\frac{g}{z}}$$ (7)

The ground height was set to $z_{Z}=0$ . The following equation of state can be obtained from Eqs 6, 7.

$$\frac{d}{dt}\left[\begin{array}{c} x\\ \dot{x} \end{array}\right]=\left[\begin{array}{cc} 0 & 1\\ \zeta^{2} & 0 \end{array}\right]\left[\begin{array}{c} x\\ \dot{x} \end{array}\right]+\left[\begin{array}{c} 0\\ -\zeta^{2} \end{array}\right]x_{ZMP}$$ (8)

When $x_{ZMP}$ is fixed to $x_{Z0}$ , it has the eigenvalues λ=(ζ,ーζ) and the corresponding eigenvectors $u={\left[\begin{array}{cc} 1 & \zeta \end{array}\right]}^{T},{\left[\begin{array}{cc} 1 & -\zeta \end{array}\right]}^{T}$ . The phase space locus has a saddle-shaped point $x_{Z0}$ and asymptotes in Eqs 9, 10.

$$x-\frac{\dot{x}}{\zeta }=x_{Z0}$$ (9)

$$x+\frac{\dot{x}}{\zeta }=x_{Z0}$$ (10)

This is considered to be equivalent to an inverted pendulum in which the whole body is composed of rods having no mass and mass.

### State Feedback Controller Modeling

The motion of an inverted pendulum is determined by the relationship between the contact point with the environment and the position of the center of gravity, but in the case of biped, the point of contact with the environment is on the ground and ZMP is the center of contact. However, since the area where ZMP exists is limited to the area where the foot is supported, it is subject to the following restrictions.

$$x_{ZMP\_min}\leq x_{ZMP}\leq x_{ZMP\_max}$$ (11)

**FIGURE 12:**
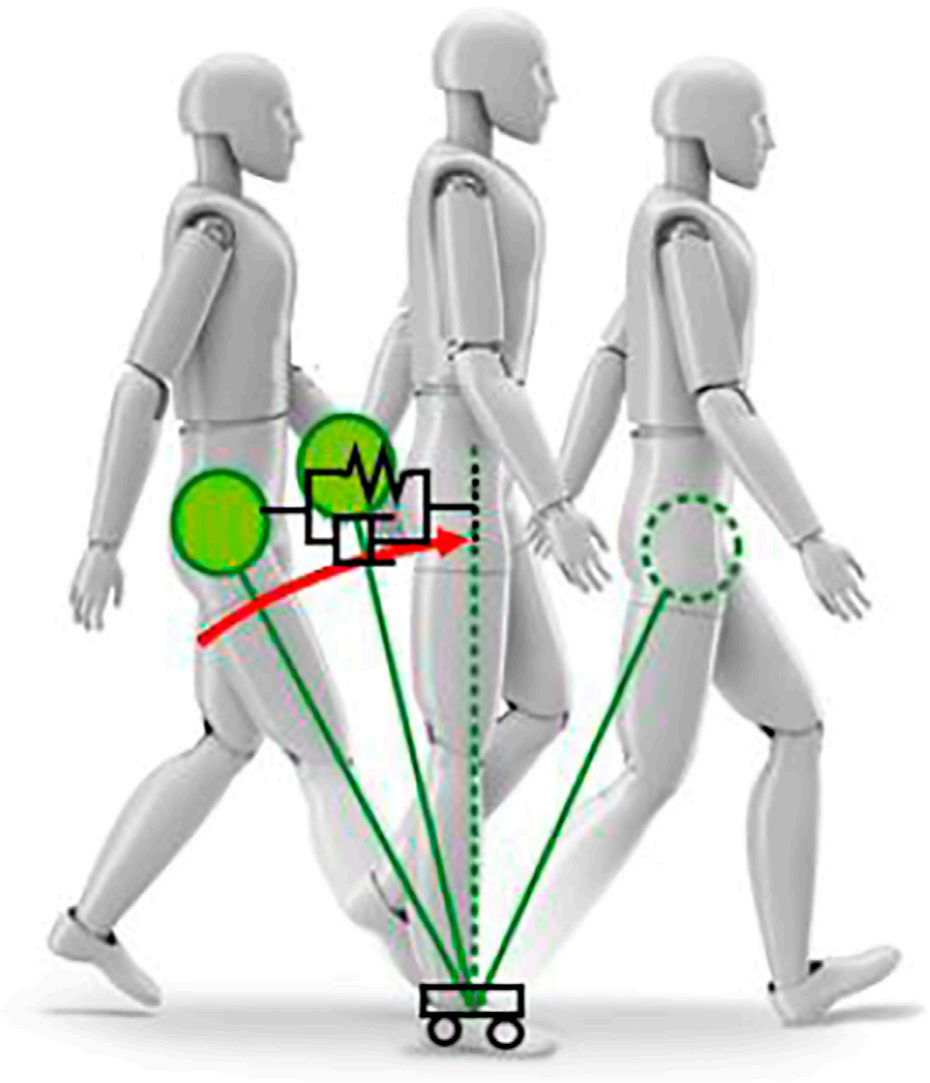
Human Balance phase based on Inverted Pendulum Model.

The state of the center of gravity can be controlled by properly manipulating the ZMP position within the support area. If there is a virtual spring damper to stabilize the center of gravity at a certain reference point, it can be understood that the spring provides the restoring force to the reference point and the damper suppresses the vibration. If the control is performed so as to kick the ground in order to obtain the effect of this restoring force, the target ${\tilde{x}}_{ZMP}^{d}$ as the manipulated variable is expressed by the following linear sum of the center of gravity position and velocity (Sugihara and Yamamoto, 2017) shown in Figure 12.

$${\tilde{x}}_{ZMP}^{d}=k_{1}\left(x-x^{d}\right)+k_{2}\dot{x}+x^{d}$$ (12)

Here, $k_{1}$ is the spring constant, $k_{2}$ is the damper coefficient, and $x^{d}$ is the center of gravity reference position. The manipulated variable ${\tilde{x}}_{ZMP}^{d}$ converges the center of gravity to a certain reference point by linear feedback control according to the state of the center of gravity position and velocity. Since the actual ZMP is constrained within the support area of both feet, if the actual ZMP follows the above target ZMP without delay, the motion of the center of gravity can be described by the following the Eqs 11, 12.

$$\ddot{x}=\{\begin{array}{cc} \zeta^{2}\left(x-x_{Zmax}\right) & {\tilde{x}}_{Z}^{d}>x_{Zmax}\;\;\;\;\;\;\;\;\;\;\;\;\;\;\;\;\;\\ \zeta^{2}(\left(1-k_{1}\right)\left(x-x^{d}\right)-k_{2}\dot{x})\;\;\;\;\; & x_{Zmin}\leq {\tilde{x}}_{Z}^{d}\leq x_{Zmax}\\ \zeta^{2}\left(x-x_{Zmin}\right) & {\tilde{x}}_{Z}^{d}<x_{Zmin\;}\;\;\;\;\;\;\;\;\;\;\;\;\;\;\;\;\; \end{array}$$ (13)

The equation of state for $x_{Zmin}\leq {\tilde{x}}_{Z}^{d}\leq x_{Zmax}$ is as follows.

$$\frac{d}{dt}\left[\begin{array}{c} x\\ \dot{x} \end{array}\right]=\left[\begin{array}{cc} 0 & 1\\ \zeta^{2}\left(1-k_{1}\right) & -\zeta^{2}k_{2} \end{array}\right]\left[\begin{array}{c} x\\ \dot{x} \end{array}\right]+\left[\begin{array}{c} 0\\ -\zeta^{2}\left(1-k_{1}\right) \end{array}\right]x^{d}$$ (14)

This system has the following eigenvalues $\lambda_{1},\lambda_{2}$ .

$$\lambda_{1}=-\zeta^{2}q_{1}$$

$$\lambda_{2}=-\zeta^{2}q_{2}$$

$$k_{1}=q_{1}q_{2}+1\;k_{2}=\frac{q_{1}+q_{2}}{\zeta }$$ (15)

Generally, the inverted pendulum model has eigenvalues λ=ζ,−ζ and corresponding eigenvectors $u={\left[\begin{array}{cc} 1 & \zeta \end{array}\right]}^{T}$ , $u={\left[\begin{array}{cc} 1 & -\zeta \end{array}\right]}^{T}$ . The divergent component given by the positive eigenvalue is the unstable mode, and the convergent component given by the negative eigenvalue is the stable mode.

## Previous Research on Identification of Standing Stability Controller

### Asymmetric Movement in the Front-Back Direction

A mathematical model of human stepping and braking control was identified through a joint project with Sugihara et al. (Kojima, 2019). The COM-ZMP model (Kajita, 2009) is often used in the field of humanoid robot engineering. The equation of motion is derived from the constraint that humanoids are not mechanically connected to the ground (Kojima, 2019), but humans are also constrained by similar conditions. The subject’s center of gravity (COM) motion trajectory and zero moment point (ZMP) were measured, and control parameters were identified. In the previous research (Kojima, 2019), Kojima et al. gave subjects an arbitrary perturbation at the end of the stepping and braking movement and verified changes in human behavior with phase plot. As a result, it has been confirmed that the phase space locus is an asymmetric locus group along the x-axis direction and has directivity in the front-back direction. It can be confirmed that the slope of the asymptote is different between the trajectory of the recovery motion when rising (converging) and the trajectory of the collapsing (diverging) motion. From this result, since the rising motion toward the reference point and the falling motion away from the reference point perform asymmetrical motions, the absolute values of the two eigenvalues are not always equal. The phase space locus has eigenvalues $\;\lambda =\zeta_{1},-\zeta_{2}$ and corresponding eigenvectors $u={\left[\begin{array}{cc} 1 & \zeta_{1} \end{array}\right]}^{T}$ , $u={\left[\begin{array}{cc} 1 & -\zeta_{2} \end{array}\right]}^{T}$ for each of the unstable mode and stable mode. Considering asymmetrical motion and applying eigenvalues $\;\lambda =\zeta_{1},-\zeta_{2}$ in the phase space locus using the diagonal matrix D whose components are the eigenvalues of the system and the matrix T whose column vector is the eigenvector, the matrix representing the system is expressed as follows (Kojima, 2019).

$$TDT^{-1}=\frac{1}{\zeta_{1}+\zeta_{2}}\left[\begin{array}{cc} 0 & 1\\ \zeta_{1} & -\zeta_{2} \end{array}\right]\left[\begin{array}{cc} \zeta_{1} & 0\\ 0 & -\zeta_{2} \end{array}\right]\left[\begin{array}{cc} \zeta_{2} & 1\\ \zeta_{1} & -1 \end{array}\right]=\left[\begin{array}{cc} 0 & 1\\ \zeta_{1}\zeta_{2} & \zeta_{1}-\zeta_{2} \end{array}\right]$$ (16)

The modified system model is shown in Eq. 14.

$$\frac{d}{dt}\left[\begin{array}{c} x\\ \dot{x} \end{array}\right]=\left[\begin{array}{cc} 0 & 1\\ \zeta_{1}\zeta_{2} & \zeta_{1}-\zeta_{2} \end{array}\right]\left[\begin{array}{c} x\\ \dot{x} \end{array}\right]+\left[\begin{array}{c} 0\\ -\zeta_{1}\zeta_{2} \end{array}\right]x^{d}$$ (17)

The equation of motion is

$$\ddot{x}=\zeta_{1}\zeta_{2}\left(x-x_{Z}\right)+\left(\zeta_{1}-\zeta_{2}\right)\dot{x}$$ (18)

Ideally, if the actual ZMP follows the above target ZMP without delay, it will be modified as follows.

$$\ddot{x}=\{\begin{array}{cc} \zeta_{1}\zeta_{2}\left(x-x_{Zmax}\right)+\left(\zeta_{1}-\zeta_{2}\right)\dot{x} & {\tilde{x}}_{Z}^{d}>x_{Zmax}\;\;\;\;\;\;\;\;\;\;\;\;\;\;\;\;\;\\ \zeta_{1}\zeta_{2}\left(1-k_{1}\right)\left(x-x^{d}\right)+\left(\zeta_{1}-\zeta_{2}-k_{2}\zeta_{1}\zeta_{2}\right)\dot{x}\;\;\;\; & x_{Zmin}\leq {\tilde{x}}_{Z}^{d}\leq x_{Zmax}\\ \zeta_{1}\zeta_{2}\left(x-x_{Zmin}\right)+\left(\zeta_{1}-\zeta_{2}\right)\dot{x} & {\tilde{x}}_{Z}^{d}<x_{Zmin\;}\;\;\;\;\;\;\;\;\;\;\;\;\;\;\;\;\; \end{array}$$ (19)

In human behavior, model predictive control is often used as the behavior is decided at each time so as to predict the state in the near future and obtain the most desirable result. Model prediction control is a type of real-time optimal control that predicts the state of the system up to the finite time future at each time for a system that changes from moment to moment, including disturbance, and optimizes at each time based on that. In the stepping and braking motion operation, control is performed while predicting the state of a finite time ahead even in the transitional period of the support area change after landing, so if the control input does not change sharply, it is possible to formulate by model prediction control. Sugihara et al. proposed model predictive control in the foot control of humanoid robots (Sugihara and Nakamura, 2006; Murai and Sugihara, 2020).

The control input is determined based on the following two points.

A. Satisfying standing stability at time T finite time ahead
B. Bringing his ZMP closer to the axle at each time,

The standing stability of A) is judged by Pratt et al. using the capture point (Pratt et al., 2006). The capture point is the point where he can finally stop the center of gravity by placing a ZMP at that point, which can be derived by the following procedure (Kojima, 2019). The following homogeneous equation is obtained from Eq. 6 by setting $\chi =x-x_{ZMP}$ .

$$\ddot{\chi }-\zeta^{2}\chi =0$$ (20)

The general solution of this equation is expressed as follows using the initial values $\chi_{0}$ and ${\dot{\chi }}_{0}$ .

$$\chi \left(t\right)=\frac{1}{2}\left(\chi_{0}+\frac{{\dot{\chi }}_{0}}{\zeta }\right)e^{\zeta t}+\frac{1}{2}\left(\chi_{0}-\frac{{\dot{\chi }}_{0}}{\zeta }\right)e^{-\zeta t}$$ (21)

Considering eigenvalues $\lambda =\zeta_{1},-\zeta_{2}$ and corresponding eigenvectors $u={\left[\begin{array}{cc} 1 & \zeta_{1} \end{array}\right]}^{T}$ , $u={\left[\begin{array}{cc} 1 & -\zeta_{2} \end{array}\right]}^{T}$ for each of the unstable mode and stable mode, we have

$$x\left(t\right)=\frac{1}{1+\zeta_{1}/\zeta_{2}}\left(x_{0}+\frac{{\dot{x}}_{0}}{\zeta_{2}}-x_{ZMP}\right)e^{\zeta_{1}t}+\frac{1}{1+\zeta_{2}/\zeta_{1}}\left(x_{0}-\frac{{\dot{x}}_{0}}{\zeta_{1}}-x_{ZMP}\right)e^{-\zeta_{2}t}+x_{ZMP}$$ (22)

$$\dot{x}\left(t\right)=\frac{1}{1+\zeta_{1}/\zeta_{2}}\left(x_{0}+\frac{{\dot{x}}_{0}}{\zeta_{2}}-x_{ZMP}\right)e^{\zeta_{1}t}-\frac{1}{1+\zeta_{2}/\zeta_{1}}\left(x_{0}-\frac{{\dot{x}}_{0}}{\zeta_{1}}-x_{ZMP}\right)e^{-\zeta_{2}t}$$ (23)

The necessary condition for the center of gravity to stop when $\dot{x}\left(t\right)=0$ after a certain period of time is that the first term of Eq. 23 becomes zero.

$$x_{0}+\frac{{\dot{x}}_{0}}{\zeta_{2}}-x_{ZMP}=0$$ (24)

At this time, $\;x_{ZMP}$ is capture point $x_{C}$ , and the following equation is defined.

$$x_{0}+\frac{{\dot{x}}_{0}}{\zeta_{2}}-x_{C}=0$$ (25)

In order to bring ZMP closer to the axis foot at each time, it is necessary to minimize the following when the axis foot position is $x_{p}$ , the current time is t, and the remaining time until landing is T.

$$min\overset{T}{\underset{t}{\int }}{\left(x_{ZMP}-x_{P}\right)}^{2}dt$$ (26)

Assuming that the target stepping position is $x_{S}^{d}$ , the condition for satisfying the standing stability at time T, which is a finite time ahead, is

$$x\left(T\right)+\frac{\dot{x}}{\zeta_{2}}\left(T\right)=x_{S}^{d}$$ (27)

$$\ddot{x}-\zeta_{1}\zeta_{2}\left(x-x_{ZMP}\right)=0$$ (28)

When solved analytically at t < T, the solution is obtained in the form of the following state feedback.

$$x_{ZMP}=\frac{2\left(x_{CP}-e^{-\zeta_{1}\left(T-t\right)}x_{SP}\right)}{1-e^{-2\zeta_{1}\left(T-t\right)}}$$ (29)

$$x_{SP}\equiv x_{S}^{d}-x_{P}$$

$$x_{CP}\equiv x+\frac{\dot{x}}{\zeta_{2}}-x_{P}$$

It can be seen that $\zeta_{1}$ is related to responsiveness and $\zeta_{2}$ is a parameter related to Capture Point. From the above, a model that can express the stepping and braking motion appropriately defined by setting the reference values $x_{P}$ and $x_{S}^{d}$ . On the other hand, the influence of the change in angular momentum around the center of gravity is not considered in the model. Here, the torque around the center of gravity calculated approximately from the movements of the mass $m_{GCi}$ of each body part and the mass center position $P_{GCi}$ is taken into consideration. Assuming that the momentum and angular momentum of each link based on the center of gravity are $P_{G}$ and $L_{G}$ , ZMP is defined by Eq. (30).

$$x_{ZMP}=\frac{Mgx-{\dot{L}}_{y}}{Mg-{\dot{P}}_{z}}=\frac{M\left\{x\left(g+\ddot{z}\right)-{\ddot{x}}_{z}\right\}-{\dot{L}}_{Gy}}{M\left(g+\ddot{z}\right)-{\dot{P}}_{Gz}}$$ (30)

M is the mass of the whole body, g is the gravitational acceleration, and x is the position of the center of gravity. The angular momentum $L_{G}$ is as follows.

$$L_{G}=\underset{i}{\sum }\left(P_{GCi}\times m_{GCi}\frac{d}{dt}P_{GCi}\right)$$ (31)

Here, if $M\left(g+\ddot{z}\right)\gg {\dot{P}}_{Gz}$ , it becomes as follows.

$$x_{ZMP}=\frac{x\left(g+\ddot{z}\right)-{\ddot{x}}_{z}}{g+\ddot{z}}ー\frac{{\dot{L}}_{Gy}}{M\left(g+\ddot{z}\right)}=x_{ZMP\_model}+c{\dot{L}}_{Gy}$$ (32)

$$c=\frac{1}{M\left(g+\ddot{z}\right)}$$ (33)

The coefficient parameter c, the torque around the center of gravity, exists as a constant value, which is a negative value after landing and is close to 0 before landing regardless of the stepping width.

### Verification of Stepping and Braking Motion

In this study, as the first step to model continuous walking, motion measurement, motion analysis, and controller identification were performed for the stepping and braking motion, which can be said to be the most basic walking motion. It is an operation that breaks the stable standing state by itself and accelerates in the direction of travel, and at the same time, moves one foot in the direction of travel and stabilizes the center of gravity after the swing leg lands. The balance control model of human stepping and braking motion identified in the previous research was reproduced with this system using the Xsens motion capture system and the M3D force plate. Subject 1 (Table 1) was measured for stepping and braking motion. From the standing position, the stepping motion was measured 10 times at a stride interval of 0.1 [m] from 0.1 [m] to 0.6 [m]. In the previous research, foot takeoff and foot landing were measured so that the foot was horizontal to the ground, but this time, in consideration of the development to continuous walking, natural takeoff and landing should be performed. bottom. Eq. 32 was modeled using the information of Xsens. The movement was measured by setting a goal for the stride length, but in order to obtain a detailed stride length, it is estimated offline from the measurement results. The foot position is $x_{P}$ and target stepping position $x_{S}^{d}$ in Eq. 29 are estimated offline from the measurement results.

The calculation of the actual ZMP for verification was derived by Eq. 34 using the data of the Xsens motion capture system and the M3D force plate. The ground reaction force value was converted to the value of the global coordinate system by the IMU sensor mounted on the M3D, and the provided data was used. When $x_{i}$ , reaction force $f_{i}$ and reaction torque $n_{i}$ (i = 1 ∼4) are measured at each of the four sensor positions, ZMP $p_{ZMP}={\left[\begin{array}{ccc} x_{ZMP} & y_{ZMP} & z_{ZMP} \end{array}\right]}^{T}$ , torque $n_{z}={\left[\begin{array}{ccc} 0 & 0 & n_{Z} \end{array}\right]}^{T}$ acting around ZMP $p_{ZMP}$ is expressed as follows.

$$x_{ZMP}=\frac{\sum_{i}^{N}\left\{-n_{iy}-\left(z_{i}-z_{zmp}\right)f_{ix}+x_{i}f_{ix}\right\}}{\sum_{i}^{N}\left\{f_{iz}\right\}}$$ (34)

**FIGURE 13:**
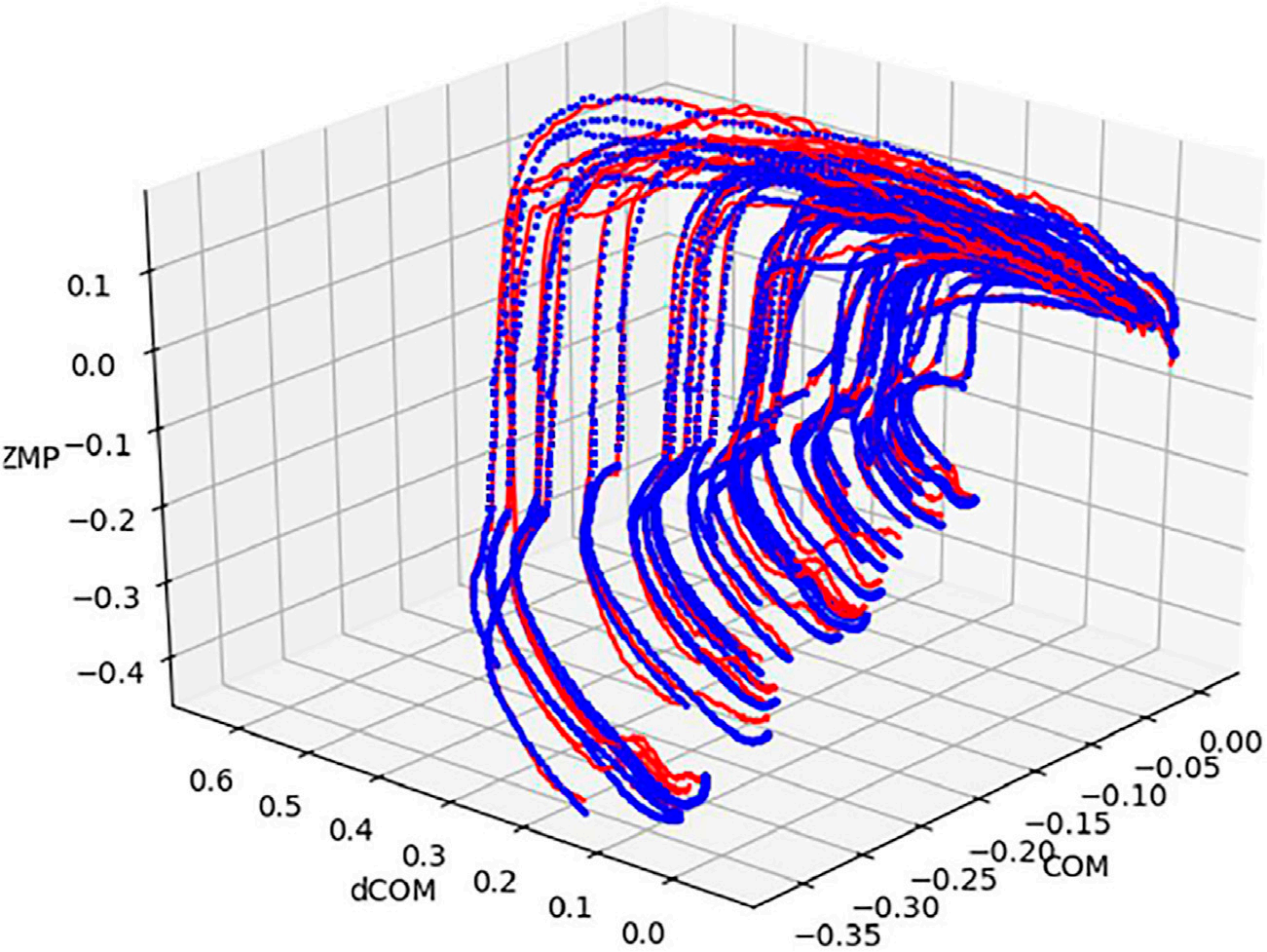
Modeled COM-dCOM-ZMP for stepping and breaking motion.

Figure 13 shows the time-series stepping and braking motion data of the center of gravity position x , velocity $\dot{x}$ , and ZMP $x_{ZMP}$ . In the figure, the red line shows the graph estimated from the sensor value, and the blue line shows the result modeled by Eq. 32. Referring the previous work with Sugihara et al., the eigenvalues $\;\lambda =\zeta_{1},-\zeta_{2}$ are constrained by $\zeta_{1}\zeta_{2}=8.67$ and the optimum value, $\zeta_{1}=4,\;\zeta_{2}=2.16$ was extracted by simulation using the data. In Figure 11, all the data are displayed with the landing position of the stepped foot aligned.

Results estimated values modeled by Eq. 32 are accurately modeled for (a) the phase up to Heel-Contact of the swing leg, (b) the phase immediately after Heel-Contact, and (c) the phase of braking the center of gravity. It was also confirmed on this measurement system that each locus after heel contact, which was confirmed in the previous research, has almost the same shape (Figure 13). Previous research results show that each locus after heel contact has almost the same shape. Therefore, it is confirmed that the measured stepping and braking motion are controlled by the same mechanism as the results of previous studies. By experimental verification of this stepping and braking motion, it was reconfirmed that if the position and velocity of the center of gravity are known by the stepping motion, the balance state before and after landing can be estimated using this model.

## Modeling Straight-Ahead Continuous Walking

### Analysis of Stepping and Breaking Motion

In the stepping and braking action performed in the previous study, the subject intentionally loses balance from a stable standing position and accelerates in the direction of travel. At the same time, by stepping one foot out in the direction of travel, the basal plane supported by both feet is expanded and returned to a stable state. The series of stepping and braking motions were modeled with a classified linear state feedback controller based on the center-of-gravity-ZMP model. In order to model simple motions, constraints were set such that the landing position and timing of the foot stepping out in the direction of travel were fixed, and the foot landed horizontally on the floor. The stepping and braking motions are combined with static elements such as walking start and walking stop, but they are not considered to contain dynamic elements necessary for modeling continuous walking. In order to construct a balance control model for continuous walking, the following points need to be considered.

A) The landing position and timing of the foot change arbitrarily
B) When landing, the landing position changes when the foot touches the ground from the heel and becomes horizontal to the floor.

**FIGURE 14:**
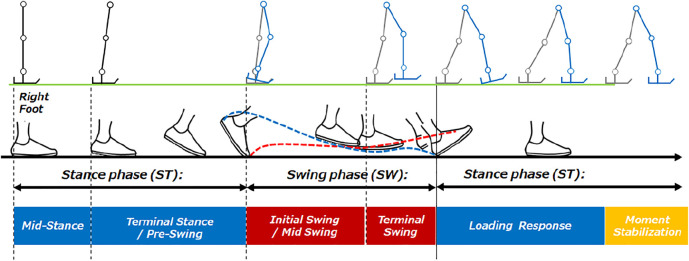
Gait analysis of stepping and braking motion.

**FIGURE 15:**
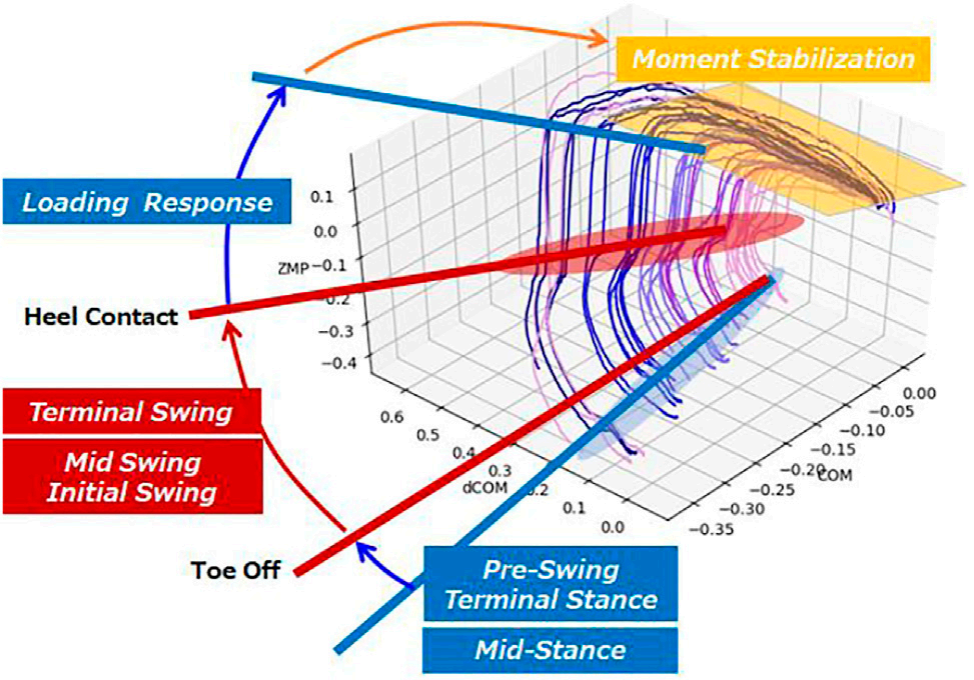
Modeled COM-dCOM-ZMP and gait phase analysis.

Figure 14 shows the result of applying the walking phase classification algorithm to stepping and braking motion. The phase of walking starts from Terminal Stance and there is no boundary between Terminal Stance and Pre-Swing. In the Swing phase, Initial Swing and Mid-Swing are activated at the same time. We have confirmed the identification of Loading Response from Terminal Swing and subsequent Heel Contact. Figure 15 shows the modeled COM-dCOM-ZMP with estimated gait phase. The characteristics of stepping and braking motion are that there is no boundary between Terminal Stance and Pre-Swing in the stance phase, and there is no boundary between Initial Swing and Mid-Swing in the Swing phase. These are typical features found at the beginning of walking. In addition, the state in which Moment Stabilization works significantly after Loading Response is a pattern peculiar to braking operation. On the other hand, in the continuous walking state, seven phases of Loading Response, Mid-Stance, Terminal Stance, Pre-Swing, Initial Swing, Mid-Swing, and Terminal Swing occur in synchronization with the left and right feet at regular intervals. Therefore, it can be considered by dividing it into three states of walking start, continuous walking, and walking stop.

### Modeling Straight-Ahead Continuous Walking

In the case of continuous walking, the first step is caused by the transition from a stationary state to a stepping motion. After the heel of the swing leg lands, it temporarily moves to the stabilization phase, but since the center of gravity accelerates in the direction of travel without any significant braking action, it moves to the destabilization phase again. At the same time, the opposite foot moves to the next step, and after the heel of the swinging leg lands, it moves to the temporary stabilization phase. The balance control model of continuous walking differs from the balance control model of the stepping and braking motion in that even if the target foot lands and brakes in a stable state, the speed of the center of gravity does not reach zero and becomes unstable again. Therefore, continuous walking is an operation established by repeating the stabilizing and destabilizing action phases. Continuous walking can be defined by the balance control model switching between the left and right foot gait phases. In this study, the left and right balance control models were constructed as follows, and switched by the gait phase of the left and right foot.

$$x_{ZMP}=\frac{2\left(x_{CP\left[RL\right]}-e^{-\zeta_{1}\left(T\left[RL\right]-t\right)}x_{SP\left[RL\right]}\right)}{1-e^{-2\zeta_{1}\left(T\left[RL\right]-t\right)}}$$

$$x_{SP\left[RL\right]}\equiv x_{S\left[RL\right]}^{d}-x_{P\left[RL\right]},x_{CP\left[RL\right]}\equiv x_{\left[RL\right]}+\frac{\dot{x\left[RL\right]}}{\zeta_{2}}-x_{P\left[RL\right]}$$ (35)

In order to switch the left and right balance control models by the timing of the gait phase of the left and right foot, the following combinations of the start and end timing of switching were considered.

A) Start timing: toe-off/end timing: heel contact
B) Start timing: toe-off/end timing: toe-off on the opposite side
C) Start timing: heel contact on the opposite side/End timing: heel contact

In this study, we applied the timing of the Gait phase estimated by the Gait phase Detection Algorithm constructed in this study using the measurement data acquired from the Xsens motion capture system and the M3D force plate, and simulated switching between the left and right balance control models. As a result, we adopted the start and end timing of B).

**FIGURE 16:**
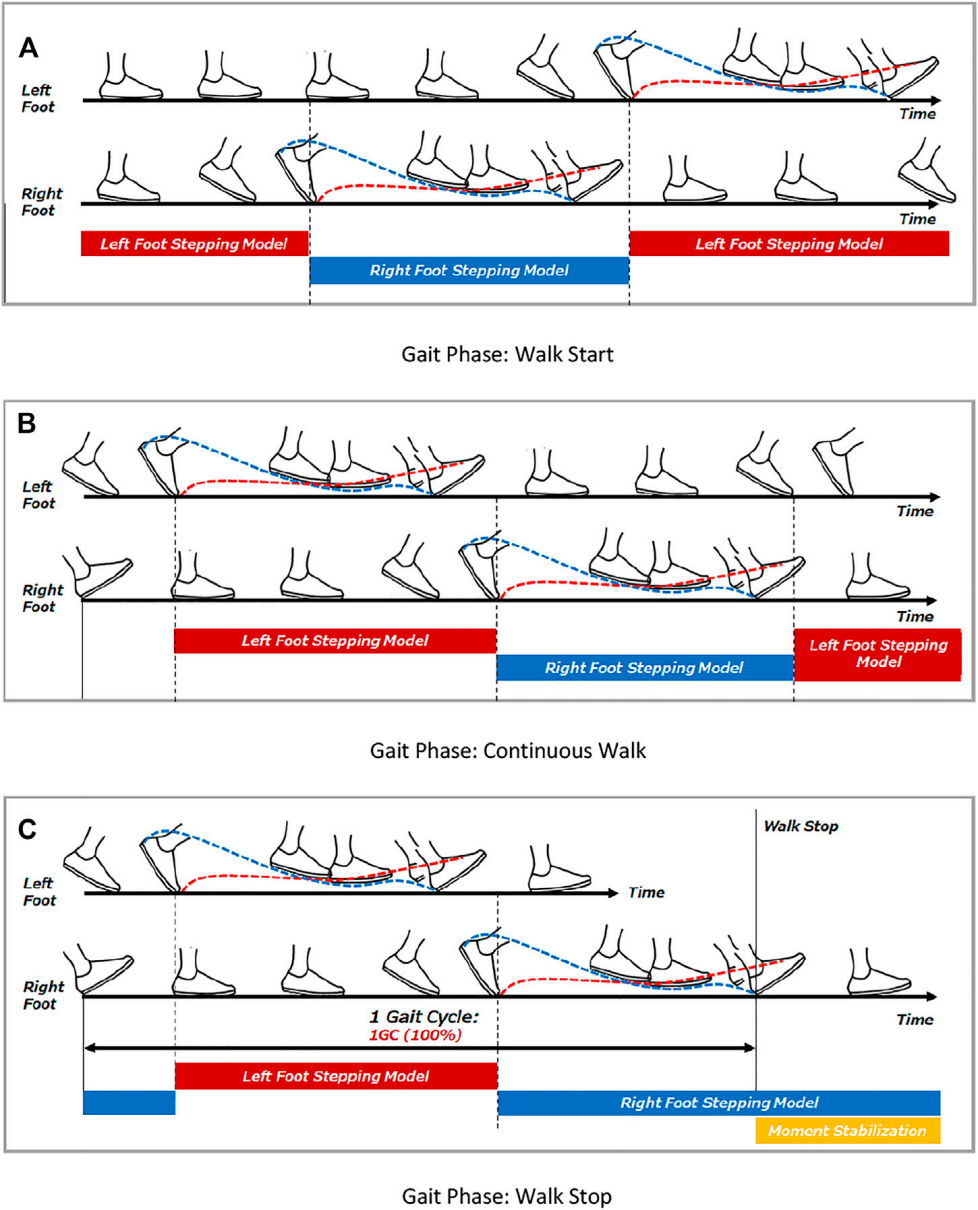
Gait timing to compute balance control model. **(A)** Gait Phase: Walk Start. **(B)** Gait Phase: Continuous Walk. **(C)** Gait Phase: Walk Stop.

**FIGURE 17:**
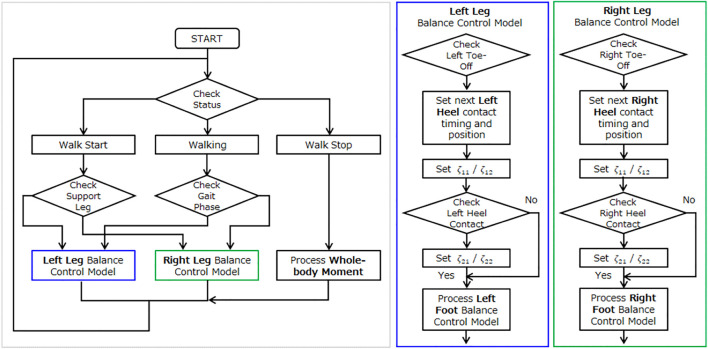
Process to generate balance control model.

Figure 16 shows the start and end timing of balance control model for each leg. Figure 17 shows the algorithm which was developed for continuous walking. The walking data (Figure 8: Walk Type 1) of subjects 1, subject 2 and subject 3 in Table 1 were used for six straight steps and 10 trials. The timing of Toe-Off was derived in advance from the measured time-series data, and the balance control model was switched at the timing of Toe-Off of the left and right feet. The timing of the next Heel Contact was also derived from the measured time series data. We divided the sections before and after the Heel Contact of the stepping foot and estimated the system parameters $\zeta_{1}$ and $\zeta_{2}$ and the control parameter $x_{P}$ in each section.

**FIGURE 18:**
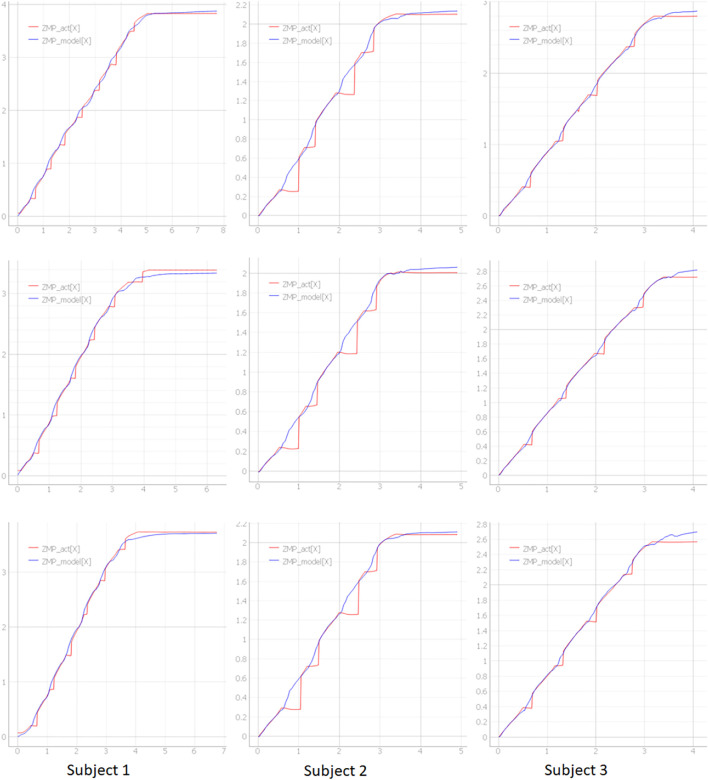
The data for each of the 3 trials for each subject.

**FIGURE 19:**
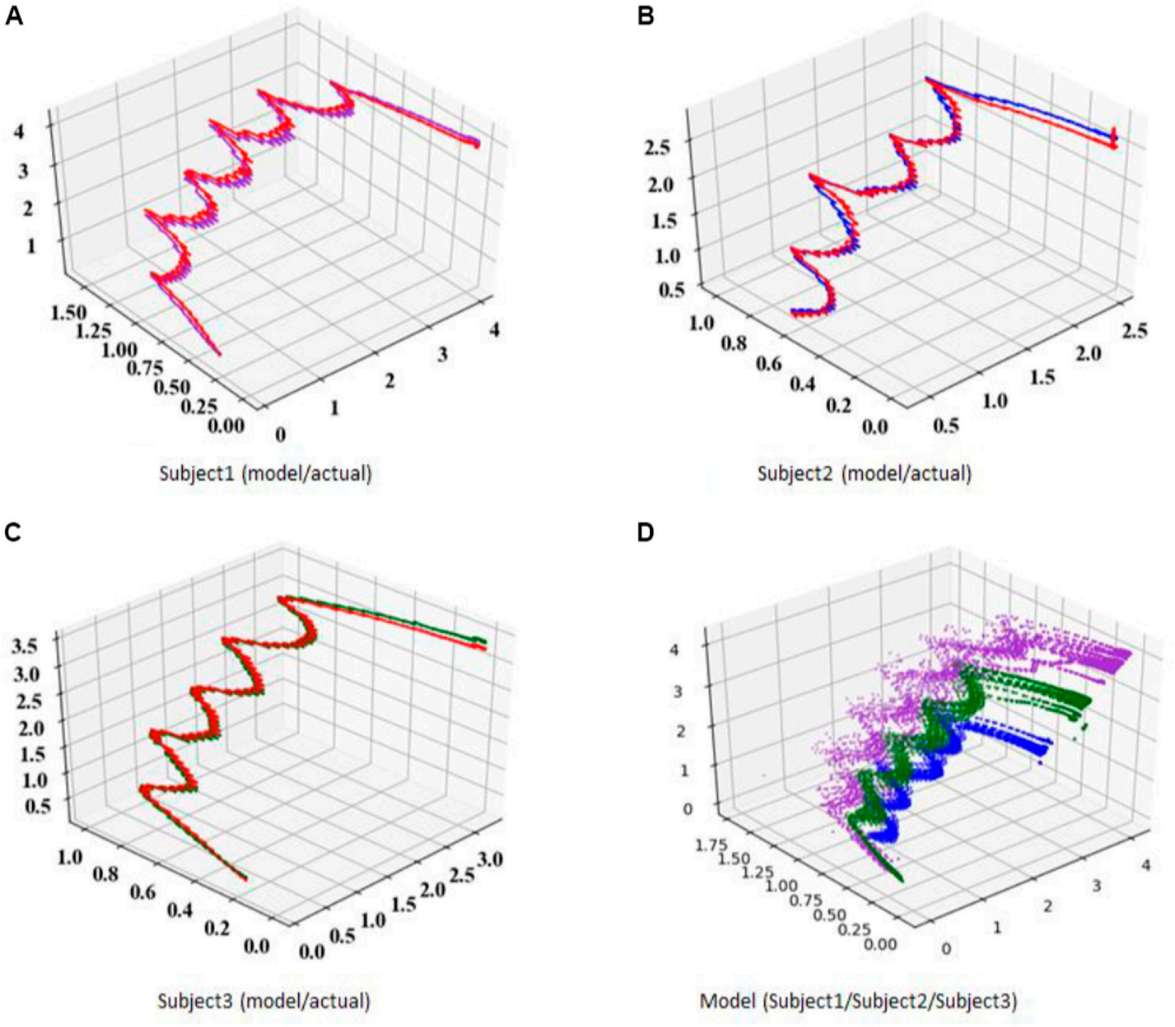
Plot of modeled COM-dCOM-ZMP for straight ahead walk. **(A)** subject1 (model/actual). **(B)** subject2 (model/actual). **(C)** subject3 (model/actual). **(D)** model (subject1/subject2/subject3).

Figure 18 shows the ZMP estimation result by the sensor (red line) and the result of the ZMP model of this method (blue line) with the three trials during the measurement of each subject’s 10 trials. Subject 1 and subject 2 are similar in body shape and height at about 1.65 [m], and subject 3 has a height of 1.80 [m] or more and a slim body shape. In Figure 18, the ZMP estimation result based on the sensor value is shown by the red line and the estimation result of the model by this method is shown by the blue line. The estimation results by the model do not consider the discontinuous behavior of ZMP due to the switching of the support legs and are displayed as continuous behavior. By wearing the measuring instrument, it is presumed that the movement of the subject 2 was not a natural movement. On the other hand, although there is a large difference in height between subject 1 and subject 3, it can be seen that walking is similar. Figures 19A–C show a plot of a modeled COM-dCOM-ZMP with N = 3. In addition, Figure 19D shows a balance control model with N = 3 and 10 trials, respectively. It can be seen that the constructed model can reproduce the ZMP estimation value by the sensor from the standing position to the walking state, the continuous walking state, and the continuous walking state to the walking stop. The model constructed by this method is effective as a model for estimating the balance control state in a straight walk state.

## Extension to Continuous Walking Including Curves

Based on the constructed balance control model, the model can also be extended to gait other than straight-ahead walking. The model extension considered the following:

A) The movement of the foot during walking can be regarded as the anteroposterior and lateral movement around the local coordinate system fixed to the trunk.
B) While walking, the swing leg side of the waist rotates in the direction of travel, and the support leg side of the waist rotates in the direction of the support legs. On the other hand, the chest above the waist generally faces the direction of travel.

Based on the above, the following preconditions were set to extend the algorithm to walking other than straight-ahead walking.

### Prerequisites

Define a local coordinate system centered on the position of the waist projected onto the ground, with the front of the chest as the X-axis and the vertical upward as the Z-axis. Build a balance control model with walking motion with the X-axis direction as the traveling direction in the local coordinate system.

**FIGURE 20:**
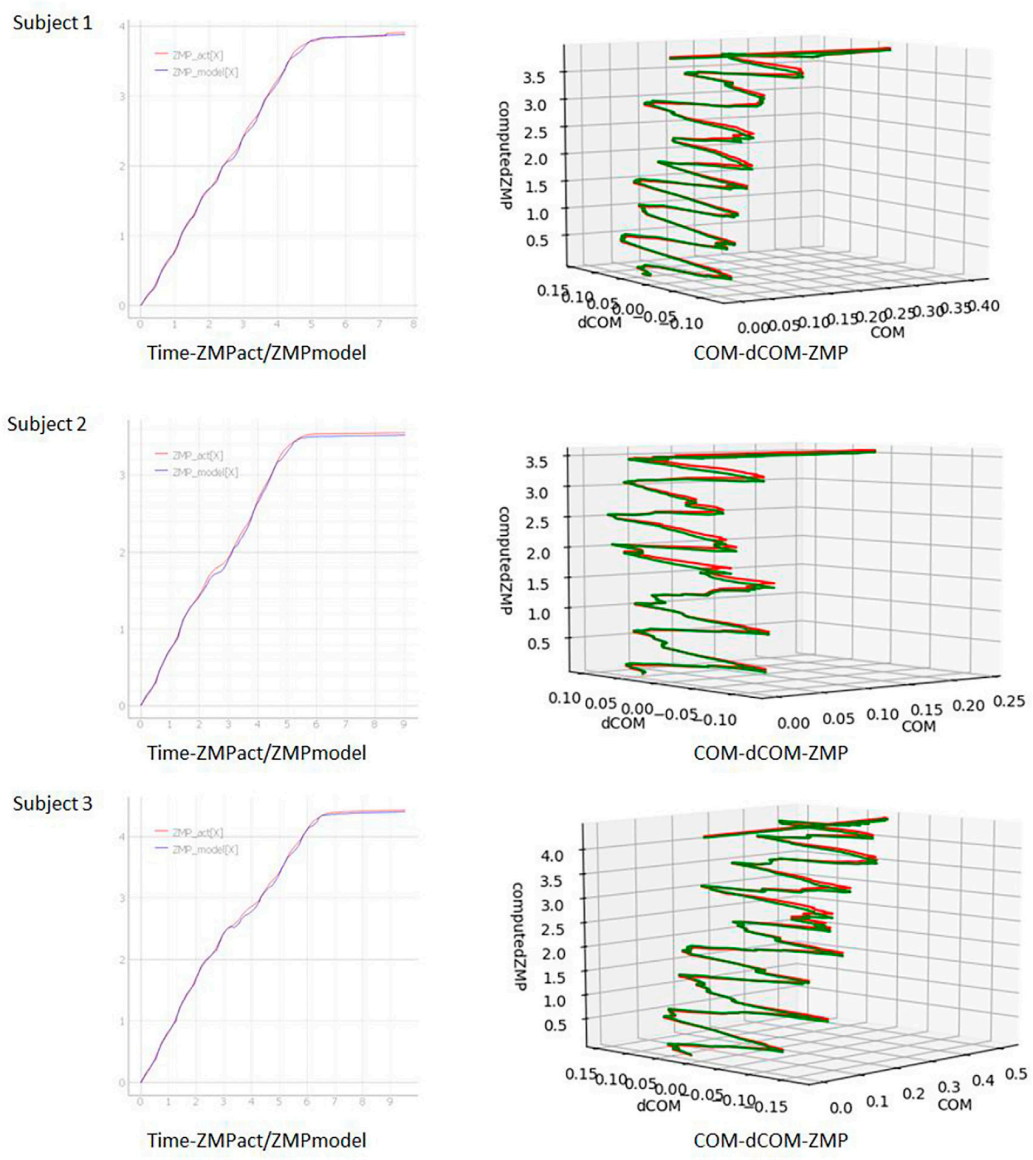
Plot of modeled COM-dCOM-ZMP for straight-90 deg turn-straight walk.

The movement of the foot during walking can be regarded as the anterior-posterior and lateral movement around the local coordinate system fixed to the trunk. In order to support walking other than straight ahead, motion data measured in the global coordinate system is converted into a local coordinate system fixed to the trunk above the waist regardless of the direction of travel, and the balance state is estimated in the local coordinate system. Regarding the balance during walking, it was considered that the change in the anteroposterior movement had a greater effect on the balance than the change in the lateral movement of the foot. For this reason, we built a balance control model that focuses only on the traveling direction (front-back direction) of the local coordinate system fixed to the trunk. Ten trials of straight-ahead, 90-degree curve, and straight-ahead line measurements were performed on the same three subjects as the straight-ahead data measurement. The measured data was converted to a local coordinate system. The algorithm of the balance control model of straight walking was converted into a local coordinate system and modeled. Figure 20 shows typical measurement results. In Figure 20, COM-dCOM were converted into the global coordinate system for visual effects. In Figure 20, the measured ZMP is shown by the red line and the model ZMP is shown by the green line. A coordinate system in the direction of travel of the global coordinate system. In each figure, it is rotated 90° to the right near the midpoint, but it can be seen that the balance control model modeled by this method can follow the measured ZMP before and after that. In continuous walking, we confirmed that the model can be expanded by targeting the balance of the direction of travel in the local coordinate system with the direction of the chest as the direction of travel.

## Summary

In this study, we constructed a balance control model for continuous walking by referring to the balance control model modeled by stepping and braking motion in the previous study.

1. In order to build a balance control model for continuous walking, we built a mobile measurement environment using the Xsens Motion Capture System and the M3D Force Plate System.
2. Using the constructed measurement environment, we measured the continuous gait of three subjects, identified the Gait phase by Perry’s Gait Analysis, and performed gait analysis.
3. Using the constructed measurement environment, verification of stepping and braking motion performed in the previous research, identification of gait phase, and gait analysis were performed.
4. From the walking analysis results of continuous walking and the walking analysis results of stepping and braking motion, the balance control model constructed by stepping and braking motion is developed at the timing of walking start, the timing of continuous walking, and the timing of walking stop. went. At the same time, the balance control model constructed by stepping and braking motion was extended to the left and right feet, and the timing of switching between the left and right models was examined.
5. The constructed balance control model for continuous walking was verified using the data of straight walking of three subjects. It was confirmed that the balance control model of continuous walking can accurately follow the ZMP estimated from the sensor in the state of starting walking, continuous walking, and stopping walking.
6. The balance control model for continuous walking in a straight line was modeled in a local coordinate system centered on the position where the waist was projected on the ground and the front direction of the chest was the X-axis direction. It was confirmed that this balance control model can be extended to the direction change during walking by modeling the behavior of the foot in the anteroposterior direction in the local coordinate system.

From the above, in continuous walking, by setting the next landing position and timing of each step, it was possible to construct a framework for modeling the balance state from before and after Toe-Off to after Heel Contact. By using this model, it is considered possible to continuously estimate whether or not the balance state is stable by comparing the deviation between the predicted balance control state and the actual balance state. It is also possible to consider a case where the deviation between the estimation of the balance control model and the actual balance state is large as a state leading to a fall. On the other hand, it is not easy to accurately set the next landing position and timing of each step. In this project, we are also predicting the next landing position and timing by machine learning, and it is a future task to build a balance control model using the prediction by machine learning (Yoshikawa, 2020).

## Data Availability

The datasets presented in this article are not readily available because Approval of company’s publication management team is required. Requests to access the datasets should be directed to taizo_yoshikawa@jp.honda.

## Footnotes

1
https://www.tecgihan.co.jp/en/products/force-plate/small-for-shoes/m3d-force-plate-wired/

2
https://www.xsens.com/
